# Potential to Avert Additional Influenza Burden in the United States with Use of Adjuvanted vs. Standard Influenza Vaccines in Individuals 50–64 Years of Age

**DOI:** 10.3390/vaccines14050380

**Published:** 2026-04-23

**Authors:** Ian McGovern, Roberto Flores, Mendel D. M. Haag

**Affiliations:** 1 CSL Seqirus, Waltham, MA 02169, USA; 2 CSL Seqirus, 08014 Barcelona, Spain; 3 CSL Seqirus, 1105 BJ Amsterdam, The Netherlands

**Keywords:** influenza, adjuvanted influenza vaccine, relative vaccine effectiveness, modeling, disease burden

## Abstract

Background: There is a high burden of influenza among individuals aged 50–64 years, with the highest rates of influenza infections other than children. The MF59-adjuvanted influenza vaccine (adjuvanted trivalent influenza vaccine [aTIV]/adjuvanted quadrivalent influenza vaccine [aQIV]) is designed to enhance response to vaccination among older adults. Among those aged ≥65 years, adjuvanted vaccine (aTIV/aQIV) has shown to be 14% more effective than standard (TIV/QIV) vaccines. This modeling study aimed to estimate the potential public health impact of aTIV/aQIV over standard influenza vaccines (TIV/QIV) among individuals aged 50–64 years over five influenza seasons. Methods: A static compartmental model was developed based on a Centers for Disease Control and Prevention model. Model inputs included vaccine effectiveness, vaccine coverage, population counts and disease burden estimates. Additional burden averted (symptomatic cases, outpatient visits, hospitalizations, intensive care unit [ICU] admissions, and deaths) was expressed as total incremental cases averted between the vaccines. Sensitivity analyses explored the influence of uncertainties in model input on the results. Results: Across the influenza seasons evaluated, on average each 5% increase in the relative vaccine effectiveness (rVE) of aTIV/aQIV vs. QIV prevented an additional 172,738 symptomatic illnesses, 74,277 outpatient visits, 1832 hospitalizations, 343 ICU admissions, and 105 deaths. This corresponds to an average seasonal incremental burden averted of 15.2%, with a range of 5.9% to 37.2%. Deterministic sensitivity analyses revealed the greatest variability was tied to rVE and burden estimates. Probabilistic sensitivity analyses results were normally distributed. Conclusions: Individuals aged 50–64 years could benefit from use of aTIV/aQIV over TIV/QIV, with an average increase in the number of influenza outcomes prevented of 15.2% per 5% improvement in vaccine effectiveness.

## 1. Introduction

Seasonal influenza poses a substantial public health burden annually. The United States (US) Centers for Disease Control and Prevention (CDC) estimated that between 2010 and 2024, influenza infections resulted in 9.3–41 million symptomatic illnesses, 120,000–710,000 hospitalizations, and 6300–52,000 deaths annually in the US [1]. Incidence of influenza varies considerably by age. A study evaluating influenza incidence between 2010 and 2016 found that incidence was highest among young children (0–4 years) with a median incidence of 13.2%, followed by adults 50–64 years of age (12.0%) [2]. While the incidence of influenza is typically lowest among older adults aged ≥65 years (3.9%), that age group has the highest rates of severe complications, accounting for 50–70% of influenza-related hospitalizations and 70–85% of influenza-related deaths [2,3].

While older adults aged ≥65 years have the highest rate of severe events, that increased risk begins before the age of 65. The process of age-related immune decline (immunosenescence) starts as young as age 20, but often begins to accelerate around the age of 50 [4]. In addition to immunosenescence, the prevalence of comorbidities that increase the risk of severe complications following an influenza infection also increase with age, with 38% of individuals aged 18–49 years having at least one high-risk condition compared with 62% for 50–64-year olds, and 80% for those aged ≥65 years [5]. This increased susceptibility to influenza is reflected in the high burden of symptomatic illnesses among individuals aged 50–64 years, as well as in hospitalization rates, which tend to stay relatively flat until around the age of 50 when hospitalization rates then begin to increase exponentially with age [6]. Individuals 50–64-years old are hospitalized for influenza at double the rate of individuals aged 18–49 years (median rate of 106 vs. 53 per 100,000 people) [6].

Influenza vaccination is the most effective method for mitigating the public health impact of influenza. The specific age and risk groups recommended for influenza vaccination vary from country to country, but young children and older adults (≥65 years) are among the most commonly targeted groups for influenza vaccination globally. Children and adults younger than 65 years of age most commonly receive the ‘standard’ egg-based non-adjuvanted influenza vaccine (trivalent influenza vaccine [TIV] or quadrivalent influenza vaccine [QIV]). However, due to age-related immunosenescence and comorbidities, ‘standard’ influenza vaccines may elicit a diminished immune response in older adults compared with younger age groups [7,8]. Adjuvanted or higher-dose influenza vaccines can help improve response to vaccination among older adults, and these vaccines are preferentially recommended for individuals aged 65 years and older in the US and many other countries [9,10,11].

Despite individuals aged 50–64 years having a high burden of influenza, they are not included (or only partially included) in the vaccination programs for many countries. Out of the 27 European Union (EU) countries, five do not offer influenza vaccine coverage for anyone 50–64 years old, and another five countries currently recommend influenza vaccination for everyone aged ≥50 years (Austria, Bulgaria, Czechia, Poland, and Romania). The remaining 17 countries either offer coverage for individuals aged 50–64 years only if patients have one or more high-risk condition (11 countries) and/or have a recommendation for adults starting below 65 years of age (12 countries, all but one of which began at 59 or 60 years instead) [12]. In the US, influenza vaccination is recommended for everyone 6 months and older [9]. Increased vaccination of individuals aged 50–64 years, as well as use of more effective influenza vaccines, can both help to reduce the burden of influenza in this age group.

A prior systematic review and meta-analysis showed that the three currently available ‘enhanced’ influenza vaccines (recombinant, adjuvanted and high-dose vaccines) were all more effective (10–19% more effective) than standard-dose, non-adjuvanted, egg-based influenza vaccines and that there were no differences observed in the effectiveness of the three enhanced vaccines [13]. Similarly, another prior systematic review found the adjuvanted vaccine to be 14% more effective compared with standard dose among adults aged ≥65 years [14]. Since 2024, the MF59-adjuvanted, inactivated influenza vaccine (adjuvanted trivalent influenza vaccine [aTIV]/adjuvanted quadrivalent influenza vaccine [aQIV]) (FLUAD^®^, Seqirus Inc., Holly Springs, NC, USA) has been approved for use in individuals aged ≥50 years in the EU and United Kingdom (UK) (but not currently the US).

The effectiveness of aTIV/aQIV has been evaluated in numerous studies among older adults aged ≥65 years [13,14,15]. The adjuvanted vaccine has also demonstrated superior immunogenicity compared with standard-dose vaccines for both H1N1 and H3N2 among individuals 50–64 years old [16]. However, as the indication of the adjuvanted vaccine for people over 50 years is recent in Europe and the UK, there is currently a lack of effectiveness data in the 50–64-years age group. This study aimed to evaluate the potential public health impact of using aTIV/aQIV instead of TIV/QIV among individuals aged 50–64 years using a modeling approach that included a range of potential relative vaccine effectiveness (rVE) values and influenza season-specific epidemiological data from the US CDC.

## 2. Materials and Methods

### 2.1. Model Design and Structure

We used a static compartmental model to estimate the hypothetical additional burden averted with use of the adjuvanted influenza vaccine compared with standard-dose influenza vaccines across five influenza seasons. The model was based on a monthly cycle length, with the influenza season defined as the period between October and September of the following year. For each influenza season assessed, two scenarios were evaluated, one in which everyone received standard-dose influenza vaccines and another where everyone received the adjuvanted influenza vaccine. Influenza burden estimates were based on season-specific data reported by the US CDC. Burden-averted estimates (symptomatic cases, outpatient visits, hospitalizations, intensive care unit [ICU] admissions and deaths) were expressed as the total cases prevented for each vaccine, as well as the incremental cases prevented between the vaccines (i.e., total averted aTIV/aQIV—total averted TIV/QIV).

The design of the model was based on a previously recommended calculation method to estimate disease burden averted by vaccinations [17]. Specifically, the model used Method 2 as per Tokars et al. [17]. The model included seven health states that were defined through combinations of patient status variables (ill [case] or well [non-case], vaccinated or nonvaccinated, and immune or susceptible) (Supplementary Figure S1). The model applied vaccine coverage and effectiveness only to the non-case population. All models were implemented in Microsoft Excel^®^.

### 2.2. Model Inputs

Vaccine effectiveness, vaccine coverage, population counts and disease burden estimates were obtained from the literature and CDC surveillance data. Data for the five influenza seasons spanning 2017–2020 and 2022–2024 influenza seasons were included in the model. The 2020–2021 and 2021–2022 influenza seasons were not evaluated due to limited influenza activity caused by the SARS-CoV-2 pandemic.

#### 2.2.1. Population Size

The population size of 62,110,000 adults 50–64 years of age was based on 2019 US census data [18] and was assumed to be constant across seasons.

#### 2.2.2. Vaccine Effectiveness

Vaccine effectiveness was defined using a combination of absolute and relative estimates. Based on the inputs available in the literature, absolute vaccine effectiveness (aVE) was derived for both vaccines. Overall (any vaccine) aVE values published by the CDC for the closest-matching age group were used as a proxy for aVE of TIV/QIV. For the 2017–2020 seasons, aVE values for 50–64 year olds were available, while the aVE estimates for the 2022–2024 seasons were only available for the broader 18–64 year age group at the time of the analysis [19]. The aVE values used are summarized in Table 1.

**Figure 1:**
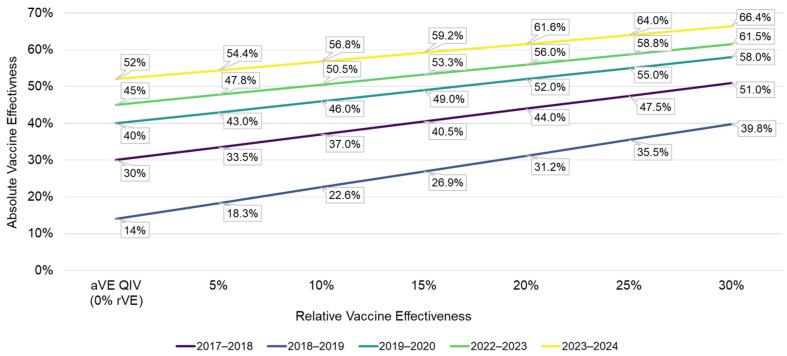
Change in aVE of aTIV/aQIV at different values of rVE for aTIV/aQIV Versus QIV. aQIV, adjuvanted quadrivalent influenza vaccine; aTIV, adjuvanted trivalent influenza vaccine; aVE, absolute vaccine effectiveness; QIV, quadrivalent influenza vaccine; rVE, relative vaccine effectiveness.

In the absence of real-world estimates of vaccine effectiveness of aTIV/QIV vs. TIV/QIV among individuals aged 50–64 years, a range of rVE values from 5 to 30% rVE were evaluated. The aVE of adjuvanted vaccine was estimated by back-calculating the aVE based on the assumed aVE of TIV/QIV and the different rVE values evaluated. The change in aVE for the adjuvanted vaccine for each 5% improvement in rVE for each season is summarized in Figure 1. The aVE of aTIV was calculated using the following equation [20]:

$$aVE_{aTIV}=\left[rVE_{aTIV\;vs\;TIV}\;\times \left(1-aVE_{TIV}\right)+aVE_{TIV}\right]$$

#### 2.2.3. Vaccine Coverage

Vaccine coverage was defined as the percentage of the total 50–64-years age group population vaccinated in each season. Seasonal monthly vaccine coverage values were extracted from the CDC Weekly Influenza Vaccination Dashboard [21]. To align the CDC vaccine coverage for each season (defined as July–May of the following year) with the season definition in the current model (October–September), the model added vaccination coverage from July to September to the count for October and assumed in the model that coverage in July and September months was the same as that reported for May (i.e., May coverage is carried forward through September). Annual vaccine coverage rates for adults aged 50–64 years are summarized in Table 1 and Supplementary Figure S2. Monthly vaccine coverage rates for the 50–64-years age group for each season are shown in Supplementary Table S1.

#### 2.2.4. Incidence Timing and Monthly Case Distribution

The monthly case distribution among individuals aged 25–64 years (closest matching age group) used in the model was derived from the seasonal weekly number of positive influenza specimens for all strains (H1N1, H3N2, B/Victoria, B/Yamagata, and unsubtyped influenza A and B) based on influenza subtyping performed by sentinel public health laboratories and published by the CDC [22]. The weekly distribution of influenza cases and the strain distribution proportions are shown in Supplementary Figures S3 and S4, respectively. Strain distributions were not included in the model (only the number of positive cases across all strains), but are presented to highlight seasonal variations in strain distribution. To obtain monthly case counts, weekly counts were mapped to the corresponding month each year. The distribution of cases each month was then calculated as the percentage of cases in a month out of the total cases for the season. Monthly case distributions over the five influenza seasons are listed in Supplementary Table S2.

#### 2.2.5. Estimates of Disease Burden of Influenza

Disease burden was estimated for the number of symptomatic cases, outpatient visits, hospitalizations, ICU visits and deaths related to influenza. Burden estimates were based data on hospitalizations obtained from the Influenza Hospitalization Surveillance Network (FluSurv-NET), which conducts population-based surveillance for laboratory-confirmed, influenza-associated hospitalizations [23]. Specifically, the CDC estimates influenza incidence in specific US hospitals and then calculates the number of influenza cases and influenza-related outpatient visits using a fixed ratio relative to the total hospitalizations [24]. The CDC estimates influenza-related deaths based on the ratio of deaths to hospitalizations in a given season [24]. The number of influenza-related ICU admissions were estimated by applying CDC estimates for the proportion of influenza hospitalizations resulting in ICU admissions to the estimates for the number of influenza hospitalizations [25]. CDC-estimated incidence rates of influenza-related symptomatic illnesses, outpatient visits, hospitalizations and deaths for adults aged 50–64 years for the different influenza seasons are shown in Supplementary Figure S5 and Supplementary Table S3.

### 2.3. Sensitivity Analyses

To evaluate the impact of uncertainty in model input parameters on model outcomes, deterministic sensitivity analyses (DSA) and probabilistic sensitivity analyses (PSA) were performed. An rVE of 15% was used in all DSA and PSAs, but the sensitivity analyses results are expected to apply to other rVE values as well. The DSA/PSAs were performed for all outcomes for the 2017–2018 season and only for the number of symptomatic cases and deaths for the remaining seasons.

DSAs used fixed upper and lower bounds (e.g., 95% confidence intervals [CIs]) to evaluate how results change at the top and bottom of expected ranges. When available, the lower and upper bounds used in the DSA were sourced from the 95% CIs reported. When no CIs were reported, the bounds were assumed to be +/−20% around the point estimate used in the base case. DSA varies one model parameter at a time and results of DSAs for each parameter were ranked based on their absolute difference and presented as a tornado diagram for the incremental outcome averted. The point estimates and ranges for parameters included in the DSA for the different influenza seasons include aVE values in Table 1 and the burden estimates in Supplementary Table S3. For the rVE values, it was assumed that rVE may range from 10 to 20% for the CI of the base case of 15% rVE.

PSAs were also performed to evaluate the impact of parameter uncertainty by repeatedly sampling from a specified distribution of input parameters. For each comparison, 1000 PSA simulations were run to generate the empirical distribution for burden averted. Results of PSAs consistently stabilized before 1000 simulations. Beta distributions were assumed for aVE of TIV/QIV, rVE of aTIV/aQIV, and ICU admission rate; for all other parameters, a normal distribution was assumed. The impact of uncertainty in multiple parameters was evaluated simultaneously, and results were presented as incremental cases averted along with quartiles.

## 3. Results

**Figure 2:**
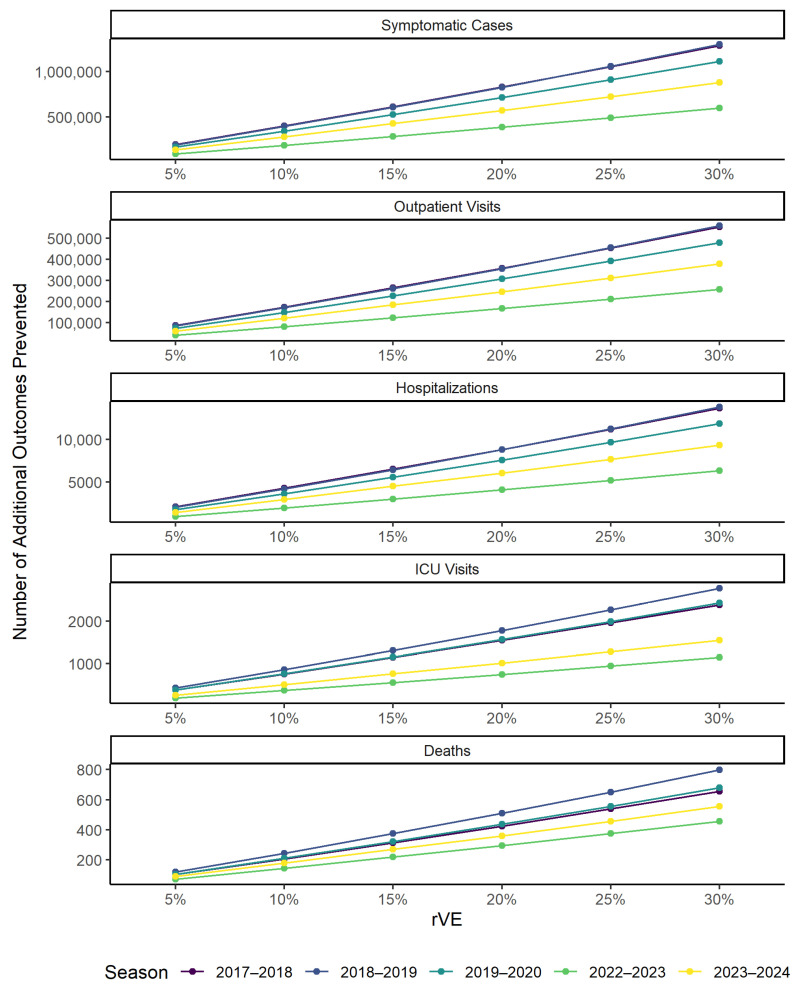
Incremental burden averted with use of aTIV/aQIV over TIV/QIV for each 5% increase in rVE. aQIV, adjuvanted quadrivalent influenza vaccine; aTIV, adjuvanted trivalent influenza vaccine; ICU, intensive care unit; QIV, quadrivalent influenza vaccine; rVE, relative vaccine effectiveness; TIV, trivalent influenza vaccine.

Across the influenza seasons evaluated, on average each 5% increase in the rVE of aTIV/aQIV vs. QIV would prevent an additional 172,738 symptomatic illnesses, 74,277 outpatient visits, 1832 hospitalizations, 343 ICU admissions and 105 deaths (Figure 2 and Supplementary Table S4). This corresponds to an average increase in burden averted of 15.2%, with a range of 5.9–37.2% (Supplementary Table S5). The largest absolute increase in the number of events prevented was seen for the 2017–2018 season, with a 5% improvement in vaccine effectiveness resulting in prevention of an additional ~200,000 symptomatic events. The smallest absolute increase was seen for the 2022–2023 season with a 5% improvement in vaccine effectiveness resulting in prevention of an additional ~93,000 symptomatic events. These observations are aligned with the 2017–2018 season having the highest and 2022–2023 having the lowest number of influenza cases out of the seasons evaluated.

In the deterministic sensitivity analyses, the underlying burden estimates and rVE estimates contributed to the most uncertainty in the model results (Supplementary Figures S6–S18). In contrast to the other model parameters evaluated in the DSA, the upper bound of the aVE estimate resulted in fewer additional cases averted while the lower bound of the 95% CI resulted in a greater number of additional cases observed. While this may seem counterintuitive, this is due to the fact that rVE represents the proportion of cases not prevented by the less effective vaccine that were additionally prevented by the more effective vaccine. In other words, as the aVE of QIV increases, a given rVE value corresponds to an increasingly smaller improvement to the aVE of aQIV. The distribution of cases from the probabilistic sensitivity analyses simulations are presented in (Supplementary Figures S19–S31). The PSA results showed that the results were all approximately normally distributed.

## 4. Discussion

There is a high burden of influenza among adults aged 50–64 years, with that age group having the highest rates of influenza infections other than children. Immunosenescence may begin to accelerate beginning around 50 years of age, contributing to potentially reduced response to vaccination along with increased susceptibility to infection. Additionally, the majority of adults aged 50–64 years have at least one high-risk condition which increases risk of severe complications like hospitalization or death following an influenza infection. Increased influenza vaccine coverage as well as the use of more effective influenza vaccines can help to reduce the burden of disease in this age group. The MF59-adjuvanted influenza vaccine (aTIV/aQIV) had its license extended down from ≥65 years in 2024 to also include use among individuals 50–64 years of age in the UK and EU. While there has not been effectiveness data generated to date, the vaccine was shown to result in superior immunogenicity compared with QIV for H1N1 and H3N2 [16].

This modeling study demonstrates how even what may appear to be a modest improvement in vaccine effectiveness (5% rVE) can have a substantial public health impact, corresponding to on average a 15.2% increase in the number of influenza cases and related complications prevented by vaccination. As a proportion of the number of cases prevented by TIV/QIV, a given rVE has largest proportionate impact when aVE is low. This is because rVE represents the proportion of cases not prevented by TIV/QIV that are additionally prevented by the more effective aTIV/aQIV. In absolute terms (i.e., how many additional cases are prevented), the benefit of a given improvement in vaccine effectiveness depends on a combination of factors including coverage levels and timing of vaccinations, timing and severity of the influenza epidemic, and vaccine effectiveness. While the added benefit is usually largest (in terms of additional cases prevented) in high-severity seasons, as was seen in this study, the benefit can vary considerably based on the other factors. For example, the 2018–2019 and 2023–2024 seasons had similar number of symptomatic cases (9,238,038 vs. 9,042,884, respectively), but a 5% improvement in vaccine effectiveness would result in approximately 30% fewer additional cases present in 2023–2024 compared with 2018–2019 (194,560 in 2018–2019 vs. 139,216 in 2023–2024). In that instance, the difference is driven primarily by differences in vaccine effectiveness between seasons, with relatively low vaccine effectiveness for 2018–2019 (14% aVE) compared with 2023–2024 (52% aVE). These results highlight how modeling approaches can be an important tool in understanding the true public health impact of influenza vaccination in a given season due to the multiple epidemiological factors that can influence the public health impact.

### Strengths and Limitations

There are strengths and limitations to this study that could influence model results. The model used the US as an example due to the availability of high-quality epidemiological data (e.g., monthly vaccination rates, burden estimates, vaccine effectiveness data) to inform the model. The study used a range of potential rVE values in assessment to provide a translation of improvements in effectiveness into public health impact without having to assume a specific rVE. The study also evaluated a range of influenza seasons, providing additional context of the observed results may vary across a range of variable season-specific epidemiological aspects (e.g., season timing/severity, vaccine performance, coverage levels).

The study also has limitations. While the study aimed to use the best available epidemiolocal data to inform the model, available data did not always perfectly align with the model requirements (e.g., influenza epidemic timing coming from 25 to 64 year olds instead of 50–64 specifically). Impact of uncertainty of model input parameters on observed results was explored in the DSA and PSA sensitivity analyses, but it cannot be excluded that more tailored input data (if it were available) could have impacted observed results. However, instances were input parameters are not perfectly aligned with the However, instances where age groups for input parameters are not perfectly aligned with the model are not expected to have drastically impacted results. While the more heterogeneous vaccination policies and availability of similar epidemiological data poses challenges to using the same modeling approach in a European context, that assessment would be more directly informative for European decision-makers. The model used a static structure that did not account for herd immunity and reduced transmission, so the model would likely underestimate the true cases averted. Overall estimates of the aVE reported by the CDC were used as a proxy for the aVE of TIV/QIV on the basis that TIV/QIV are the most commonly used vaccine among 50–64-year-olds. However, in reality, those aVE estimates likely also included individuals vaccinated with cell-based or recombinant influenza vaccines. The disease burden estimates reported by the CDC included in the model correspond to the overall population inclusive of both vaccinated and nonvaccinated individuals. Therefore, the influenza burden model parameters used are likely an underestimation of the true disease burden that would be expected in a fully unvaccinated population, resulting in a likely underestimation of true additional cases that would be averted. While the rVE values used in this study are in the range of effects seen in observational studies comparing aTIV/aQIV to TIV/QIV among individuals aged ≥65 years, future assessments would benefit from the availability of rVE data specific to the 50–64-year-old population. Finally, age-specific data were not available for the ICU admission rates and therefore assumed rates may not fully reflect the rates among 50–64-year-olds specifically.

## 5. Conclusions

Individuals 50–64 years of age have a high burden of influenza and could benefit from the use of aTIV/aQIV over QIV, with each 5% improvement in vaccine effectiveness resulting in a substantial public health benefit via an average increase of 15.2% in the number of influenza outcomes prevented.

## Figures and Tables

**Table 1:** Summary of model inputs.

Season	Vaccine Coverage ^a^	aVE ^a^ (95% CI)	Outcome	Total Events (Without Vaccination) ^b^
2017–2018	39.7%	30% (13–44%)	Symptomatic cases Outpatient visits Hospitalizations ICU visits Deaths	13,237,932 5,692,311 140,385 24,567 6751
2018–2019	47.3%	14% (0–33%) ^c^	Symptomatic cases Outpatient visits Hospitalizations ICU visits Deaths	9,238,038 3,972,356 97,967 19,789 5676
2019–2020	50.6%	40% (22–54%)	Symptomatic cases Outpatient visits Hospitalizations ICU visits Deaths	8,416,702 3,619,182 89,257 18,387 5133
2022–2023	50.1%	45% (41–48%) ^d^	Symptomatic cases Outpatient visits Hospitalizations ICU visits Deaths	6,384,316 2,745,256 67,704 12,119 4845
2023–2024	46.2%	52% (50–55%) ^d^	Symptomatic cases Outpatient visits Hospitalizations ICU visits Deaths	9,042,884 3,888,440 95,897 15,919 5701

^a^ Vaccine coverage and absolute vaccine effectiveness (aVE) estimates correspond to any influenza vaccine type approved for that age group (i.e., standard-dose non-adjuvanted vaccines, cell-based vaccines, and recombinant vaccines), but is anticipated to be primarily standard-dose non-adjuvanted vaccines. ^b^ Burden values taken from CDC estimates including both vaccinated and unvaccinated individuals, but assumed to be burden among unvaccinated for model purpose. ^c^ Reported 95% CI was −10 to 33%, but CI lower limits truncated at 0% for the model. ^d^ VISION (outpatient) estimate for individuals aged 18–64 years old. aVE, absolute vaccine effectiveness; CDC, Centers for Disease Control and Prevention; CI, confidence interval; ICU, intensive care unit.

## Data Availability

This study utilized entirely publicly available data from sources noted within in-text citations.

## Supplementary Materials

The following supporting information can be downloaded at: https://www.mdpi.com/article/10.3390/vaccines14050380/s1, Figure S1: Generalized Structure for the Reference Model; Figure S2: Vaccine Coverage 50–64-Year-Olds; Figure S3: Case Distribution by Season Among Individuals Aged 25–64 Years; Figure S4: Strain Distribution by Season, Individuals Aged 50–64 Years; Figure S5: Influenza Burden Estimates by Season; Figure S6: 2017–2018 DSA Symptomatic Cases; Figure S7: 2017–2018 DSA Outpatient Visits; Figure S8: 2017–2018 DSA Hospitalizations; Figure S9: 2017–2018 DSA ICU Admissions; Figure S10: 2017–2018 DSA Deaths; Figure S11: 2018–2019 DSA Symptomatic Illnesses; Figure S12: 2018–2019 DSA Deaths; Figure S13: 2019–2020 DSA Symptomatic Illnesses; Figure S14: 2019–2020 DSA Deaths; Figure S15: 2022–2023 DSA Symptomatic Illnesses; Figure S16: 2022–2023 DSA Deaths; Figure S17: 2023–2024 DSA Symptomatic Illnesses; Figure S18: 2023–2024 DSA Deaths; Figure S19: 2017–2018 PSA Symptomatic Cases; Figure S20: 2017–2018 PSA Outpatient Visits; Figure S21: 2017–2018 PSA Hospitalizations; Figure S22: 2017–2018 PSA ICU Admissions; Figure S23: 2017–2018 PSA Deaths; Figure S24: 2018–2019 PSA Symptomatic Illnesses; Figure S25: 2018–2019 PSA Deaths; Figure S26: 2019–2020 PSA Symptomatic Illnesses; Figure S27: 2019–2020 PSA Deaths; Figure S28: 2022–2023 PSA Symptomatic Illnesses; Figure S29: 2022–2023 PSA Deaths; Figure S30: 2023–2024 PSA Symptomatic Illnesses; Figure S31: 2023–2024 PSA Deaths; Table S1: Influenza Vaccination Rates by Month Among Individuals Aged 50–64 Years; Table S2: Distribution of Influenza Cases by Month, Based on Data from Individuals Aged 25–64 Years; Table S3: Burden Estimate and Credible Intervals by Season; Table S4: Summary of Burden Averted by Season and rVE Value; Table S5: Additional Burden Averted as a Proportion of the Total Burden Averted by TIV/QIV.

## Abbreviations

The following abbreviations are used in this manuscript:

aQIV	Adjuvanted quadrivalent influenza vaccine
aTIV	Adjuvanted trivalent influenza vaccine
aVE	Absolute vaccine effectiveness
CDC	Centers for Disease Control and Prevention
CI	Confidence interval
DSA	Deterministic sensitivity analysis
ICU	Intensive care unit
PSA	Probabilistic sensitivity analysis
QIV	Quadrivalent influenza vaccine
rVE	Relative vaccine effectiveness
TIV	Trivalent influenza vaccine
