# Supplementary material for: Potential to Avert Additional Influenza Burden in the United States with Use of Adjuvanted vs. Standard Influenza Vaccines in Individuals 50–64 Years of Age
Source: Vaccines (Basel). 2026 Apr 23;14(5):380. doi: 10.3390/vaccines14050380 (PMC13211371; doi:10.3390/vaccines14050380)
Supplement: Supplementary file 1 [file vaccines-14-00380-s001.zip › vaccines-4250381-supplementary.pdf]

Potential to Avert Additional Influenza Burden in the United States With Use of Adjuvanted vs Standard Influenza Vaccines in Individuals 50–64 Years of Age

Ian McGovern, Roberto Flores, Mendel Haag

Supplementary Materials

Supplementary Figure S1. Generalized Structure for the Reference Model

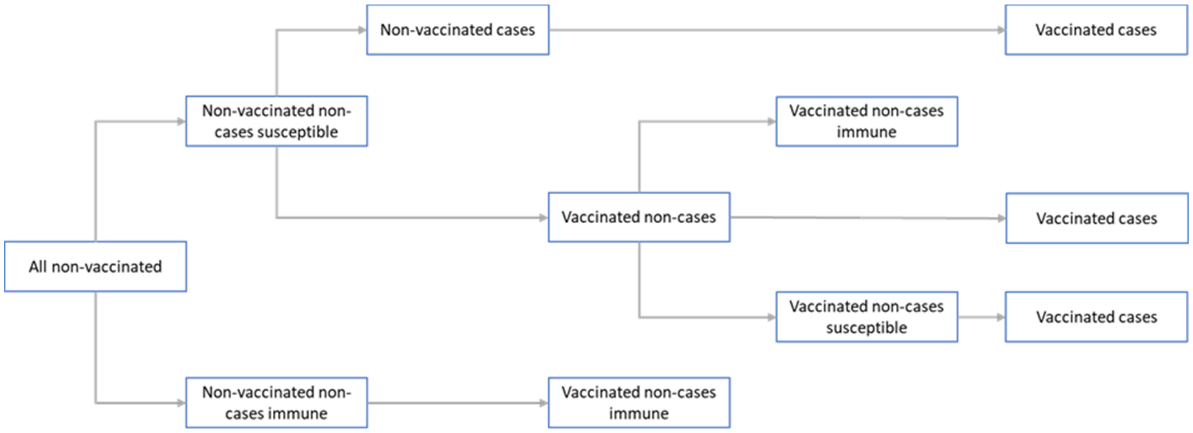

Supplementary Figure S2. Vaccine Coverage 50–64-Year-Olds

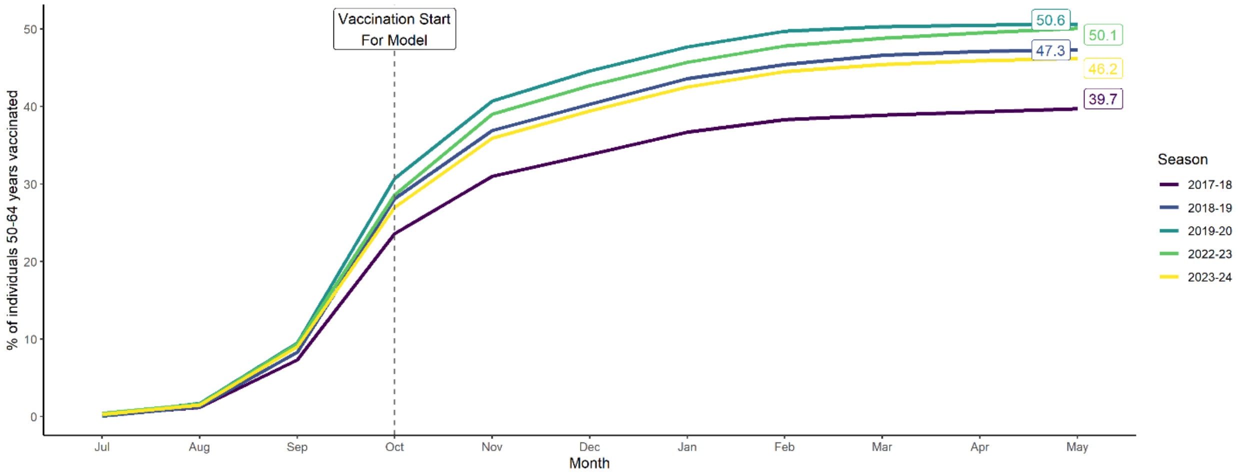

Supplementary Table S1. Influenza Vaccination Rates by Month Among Individuals Aged 50–64 Years

	Season				
**Month**	**2017–2018**	**2018–2019**	**2019–2020**	**2022–2023**	**2023–2024**
**October**	23.6%	28.1%	30.7%	28.6%	27.0%
**November**	31.0%	36.9%	40.7%	39.0%	35.9%
**December**	33.8%	40.3%	44.6%	42.7%	39.4%
**January**	36.7%	43.6%	47.7%	45.7%	42.5%
**February**	38.3%	45.4%	49.7%	47.8%	44.5%
**March**	38.9%	46.6%	50.3%	48.8%	45.4%
**April**	39.3%	47.1%	50.5%	49.5%	45.9%
**May**	39.7%	47.3%	50.6%	50.1%	46.2%
**June**	39.7%	47.3%	50.6%	50.1%	46.2%
**July**	39.7%	47.3%	50.6%	50.1%	46.2%
**August**	39.7%	47.3%	50.6%	50.1%	46.2%
**September**	39.7%	47.3%	50.6%	50.1%	46.2%

Supplementary Figure S3. Case Distribution by Season Among Individuals Aged 25–64 Years

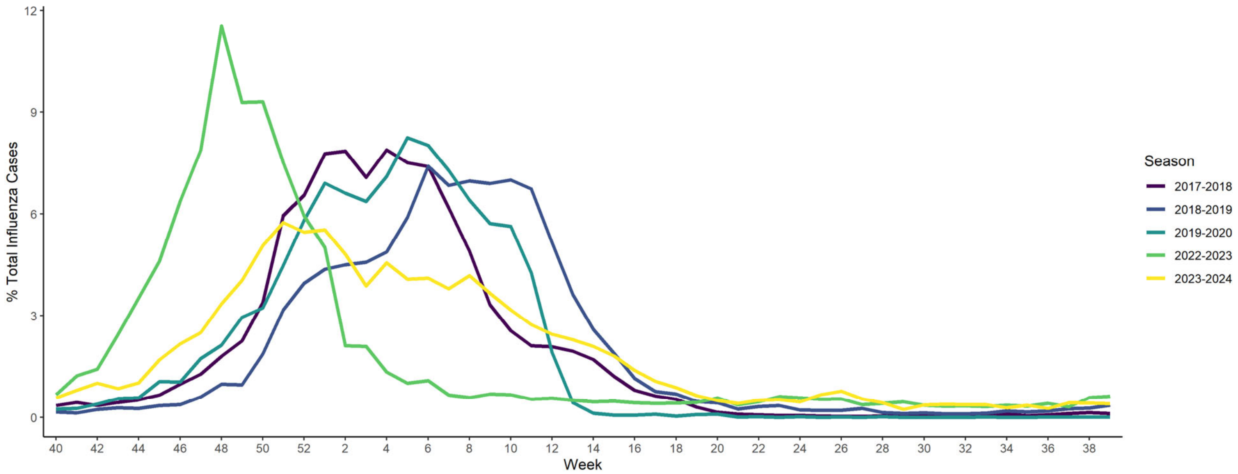

Supplementary Figure S4. Strain Distribution by Season, Individuals Aged 50–64 Years

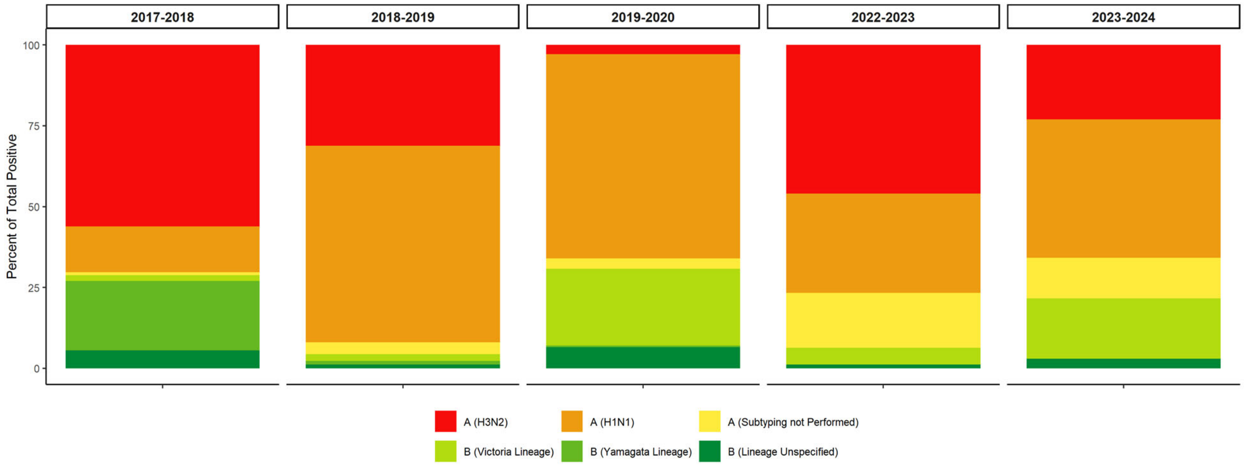

Supplementary Table S2. Distribution of Influenza Cases by Month, Based on Data from Individuals Aged 25–64 Years

	Season				
**Month**	**2017–2018**	**2018–2019**	**2019–2020**	**2022–2023**	**2023–2024**
**October**	2.1%	1.1%	2.0%	9.3%	4.2%
**November**	4.7%	2.3%	6.0%	30.4%	9.7%
**December**	18.1%	9.9%	16.5%	32.0%	20.3%
**January**	38.1%	24.2%	35.2%	11.6%	22.8%
**February**	21.8%	28.1%	27.4%	3.0%	15.7%
**March**	8.7%	22.5%	12.2%	2.3%	10.7%
**April**	4.9%	7.1%	0.4%	2.2%	7.2%
**May**	0.6%	1.5%	0.2%	1.8%	2.1%
**June**	0.2%	1.0%	0.0%	2.3%	2.4%
**July**	0.2%	0.7%	0.0%	1.9%	2.0%
**August**	0.2%	0.6%	0.0%	1.3%	1.4%
**September**	0.4%	1.1%	0.0%	1.9%	1.5%

Supplementary Figure S5. Influenza Burden Estimates by Season

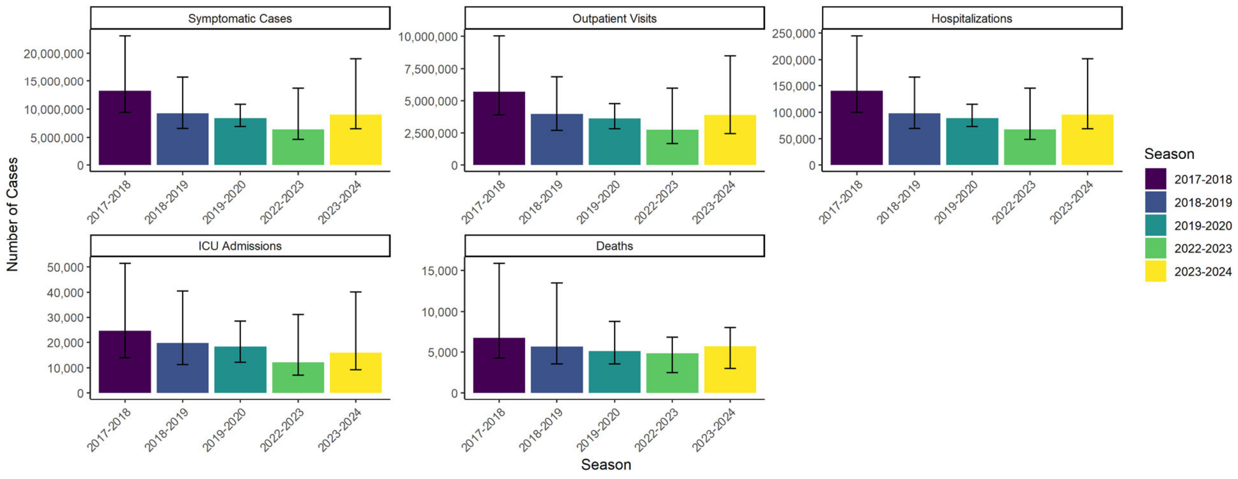

ICU, intensive care unit.

Supplementary Table S3. Burden Estimate and Credible Intervals by Season

Burden Estimate	Season				
	**2017–2018**	**2018–2019**	**2019–2020**	**2022–2023**	**2023–2024**
**Symptomatic Cases**	13,237,932 (9,400,614–23,062,957)	9,238,038 (6,582,690–15,759,286)	8,416,702 (6,918,420–10,835,295)	6,384,316 (4,613,653–13,690,632)	9,042,884 (6,536,493–19,011,230)
**Outpatient Visits**	5,692,311 (3,895,925–10,028,080)	3,972,356 (2,712,868–6,886,487)	3,619,182 (2,832,003–4,780,110)	2,745,256 (1,699,678–5,955,664)	3,888,440 (2,457,484–8,502,440)
**Hospitalizations**	140,385 (99,691–244,576)	97,967 (69,808–167,123)	89,257 (73,368–114,905)	67,704 (48,927–145,185)	95,897 (69,318–201,609)
**ICU Rate**	17.5% (14–21)	20.2% (16.2–24.2)	20.6% (16.5–24.7)	17.9% (14.3–21.5)	16.6% (13.3–19.9)
**ICU Admissions**	24,567 (13,957–51,361)	19,789 (11,281–40,511)	18,387 (12,091–28,405)	12,119 (7006–31,186)	15,919 (9205–40,161)
**Deaths**	6751 (4244–15,863)	5676 (3547–13,486)	5133 (3534–8750)	4845 (2511–6807)	5701 (3001–7973)

ICU, intensive care unit.

Supplementary Table S4. Summary of Burden Averted by Season and rVE Value

				5% rVE		10% rVE		15% rVE	
**Season**	**Outcome**	**Total Events** (Without Vaccination)	**QIV -** Events Prevented	**aTIV/aQIV -** Events Prevented	**Incremental** (QIV - aTIV/aQIV)	**aTIV/aQIV -** Events Prevented	**Incremental** (QIV - aTIV/aQIV)	**aTIV/aQIV -** Events Prevented	**Incremental** (QIV - aTIV/aQIV)
**2017–2018**	**Symptomatic Cases**	13,237,932	1,504,132	1,703,317	199,184	1,908,224	404,092	2,119,113	614,981
	**Outpatient Visits**	5,692,311	646,777	732,426	85,649	820,536	173,759	911,219	264,442
	**Hospitalizations**	140,385	15,951	18,063	2112	20,236	4285	22,473	6522
	**ICU Visits**	24,567	2791	3161	370	3541	750	3933	1141
	**Deaths**	6751	767	869	102	973	206	1081	314
**2018–2019**	**Symptomatic Cases**	9,238,038	583,040	777,600	194,560	980,242	397,202	1,191,489	608,450
	**Outpatient Visits**	3,972,356	250,707	334,368	83,661	421,504	170,797	512,340	261,633
	**Hospitalizations**	97,967	6183	8246	2063	10,395	4212	12,635	6452
	**ICU Visits**	19,789	1249	1666	417	2100	851	2552	1303
	**Deaths**	5676	358	478	120	602	244	732	374
**2019–2020**	**Symptomatic Cases**	8,416,702	1,815,674	1,985,441	169,766	2,161,181	345,506	2,343,223	527,548
	**Outpatient Visits**	3,619,182	780,740	853,739	72,999	929,308	148,568	1,007,586	226,846
	**Hospitalizations**	89,257	19,255	21,055	1800	22,919	3664	24,849	5595
	**ICU Visits**	18,387	3966	4337	371	4721	755	5119	1152

				5% rVE		10% rVE		15% rVE	
**Season**	**Outcome**	**Total Events** (Without Vaccination)	**QIV -** Events Prevented	**aTIV/aQIV -** Events Prevented	**Incremental** (QIV - aTIV/aQIV)	**aTIV/aQIV -** Events Prevented	**Incremental** (QIV - aTIV/aQIV)	**aTIV/aQIV -** Events Prevented	**Incremental** (QIV - aTIV/aQIV)
	**Deaths**	5133	1107	1211	104	1318	211	1429	322
**2022–2023**	**Symptomatic Cases**	6,384,316	1,222,513	1,315,407	92,894	1,410,980	188,467	1,509,357	286,844
	**Outpatient Visits**	2,745,256	525,681	565,625	39,945	606,722	81,041	649,024	123,343
	**Hospitalizations**	67,704	12,964	13,950	985	14,963	1999	16,006	3042
	**ICU Visits**	12,119	2321	2497	176	2678	358	2865	545
	**Deaths**	4845	928	998	70	1071	143	1145	218
**2023–2034**	**Symptomatic Cases**	9,042,884	2,341,737	2,480,953	139,216	2,623,777	282,040	2,770,355	428,618
	**Outpatient Visits**	3,888,440	1,006,947	1,066,810	59,863	1,128,224	121,277	1,191,252	184,306
	**Hospitalizations**	95,897	24,833	26,310	1476	27,824	2991	29,379	4545
	**ICU Visits**	15,919	4122	4367	245	4619	496	4877	755
	**Deaths**	5701	1476	1564	88	1654	178	1747	270

Season	Outcome	Total Events (Without Vaccination)	QIV - Events Prevented	20% rVE		25% rVE		30% rVE	
				**aTIV/aQIV -** Events Prevented	**Incremental** (QIV - aTIV/aQIV)	**aTIV/aQIV -** Events Prevented	**Incremental** (QIV - aTIV/aQIV)	**aTIV/aQIV -** Events Prevented	**Incremental** (QIV - aTIV/aQIV)
**2017–2018**	**Symptomatic Cases**	13,237,932	1,504,132	2,336,257	832,125	2,559,948	1,055,815	2,790,495	1,286,363
	**Outpatient Visits**	5,692,311	646,777	1,004,591	357,814	1,100,778	454,001	1,199,913	553,136
	**Hospitalizations**	140,385	15,951	24,775	8824	27,148	11,197	29,593	13,642
	**ICU Visits**	24,567	2791	4336	1544	4751	1959	5179	2387
	**Deaths**	6751	767	1191	424	1306	538	1423	656
**2018–2019**	**Symptomatic Cases**	9,238,038	583,040	1,411,912	828,872	1,642,131	1,059,091	1,882,827	1,299,788
	**Outpatient Visits**	3,972,356	250,707	607,122	356,415	706,116	455,409	809,616	558,909
	**Hospitalizations**	97,967	6183	14,973	8790	17,414	11,231	19,967	13,784
	**ICU Visits**	19,789	1249	3025	1776	3518	2269	4033	2784
	**Deaths**	5676	358	868	509	1009	651	1157	799
**2019–2020**	**Symptomatic Cases**	8,416,702	1,815,674	2,531,920	716,246	2,727,654	911,980	2,930,835	1,115,161
	**Outpatient Visits**	3,619,182	780,740	1,088,726	307,986	1,172,891	392,151	1,260,259	479,519
	**Hospitalizations**	89,257	19,255	26,850	7596	28,926	9671	31,081	11,826
	**ICU Visits**	18,387	3966	5531	1565	5959	1992	6403	2436

				20% rVE		25% rVE		30% rVE	
**Season**	**Outcome**	**Total Events** (Without Vaccination)	**QIV -** Events Prevented	**aTIV/aQIV -** Events Prevented	**Incremental** (QIV - aTIV/aQIV)	**aTIV/aQIV -** Events Prevented	**Incremental** (QIV - aTIV/aQIV)	**aTIV/aQIV -** Events Prevented	**Incremental** (QIV - aTIV/aQIV)
	**Deaths**	5133	1107	1544	437	1663	556	1787	680
**2022–2023**	**Symptomatic Cases**	6,384,316	1,222,513	1,610,672	388,159	1,715,068	492,555	1,822,699	600,186
	**Outpatient Visits**	2,745,256	525,681	692,589	166,908	737,479	211,799	783,760	258,080
	**Hospitalizations**	67,704	12,964	17,081	4116	18,188	5223	19,329	6365
	**ICU Visits**	12,119	2321	3057	737	3256	935	3460	1139
	**Deaths**	4845	928	1222	295	1302	374	1383	455
**2023–2034**	**Symptomatic Cases**	9,042,884	2,341,737	2,867,994	571,048	3,020,642	723,697	3,177,592	880,647
	**Outpatient Visits**	3,888,440	1,006,947	1,233,237	245,551	1,298,876	311,190	1,366,365	378,678
	**Hospitalizations**	95,897	24,833	30,414	6056	32,033	7675	33,697	9339
	**ICU Visits**	15,919	4122	5049	1005	5317	1274	5594	1550
	**Deaths**	5701	1476	1808	360	1904	456	2003	555

aQIV, adjuvanted quadrivalent influenza vaccine; aTIV, adjuvanted trivalent influenza vaccine; ICU, intensive care unit; QIV, quadrivalent influenza vaccine; rVE, relative vaccine effectiveness.

Supplementary Table S5. Additional Burden Averted as a Proportion of the Total Burden Averted by TIV/QIV

Season	% Increase in Burden Averted for Given rVE					
	**5%**	**10%**	**15%**	**20%**	**25%**	**30%**
**2017–2018**	13%	27%	41%	55%	70%	86%
**2018–2019**	33%	68%	104%	142%	182%	223%
**2019–2020**	9%	19%	29%	39%	50%	61%
**2022–2023**	8%	15%	23%	32%	40%	49%
**2023–2034**	6%	12%	18%	22%	29%	36%

QIV, quadrivalent influenza vaccine; rVE, relative vaccine effectiveness; TIV, trivalent influenza vaccine.

Supplementary Figure S6. 2017–2018 DSA Symptomatic Cases

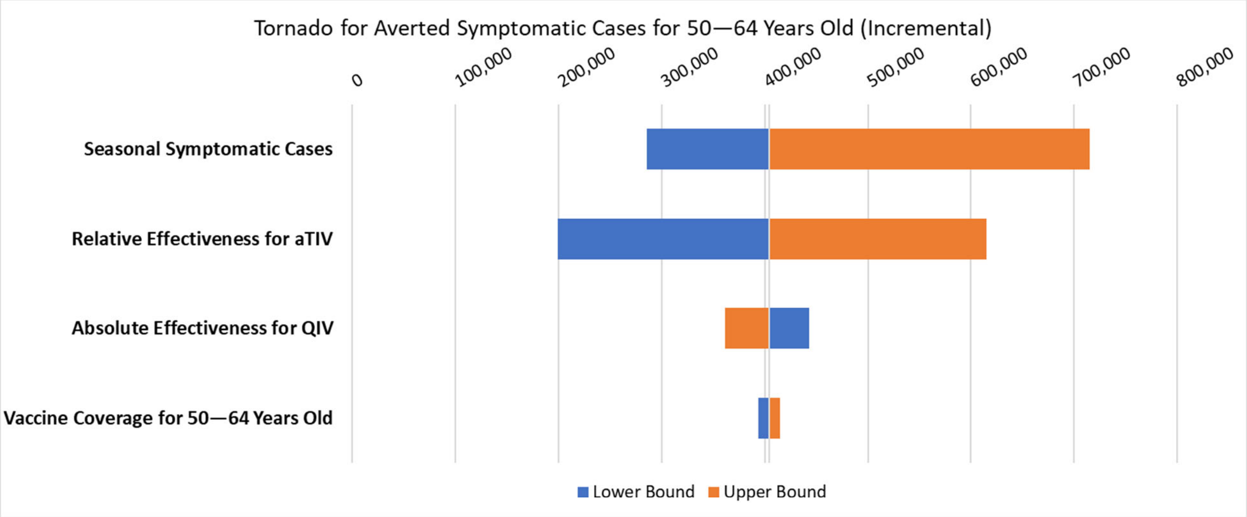

Rank	Parameter Name	Lower Bound: Symptomatic Cases Averted	Upper Bound: Symptomatic Cases Averted	Lower Bound: Percentage Change From Base Case	Upper Bound: Percentage Change From Base Case
	Base Case	404,092			
1	Seasonal Symptomatic Cases	285,375	715,278	-29.4%	77.0%
2	Relative Effectiveness for aTIV	199,184	614,981	-50.7%	52.2%
3	Absolute Effectiveness for QIV	442,668	361,175	9.5%	-10.6%
4	Vaccine Coverage for 50—64 Years Old	393,933	414,358	-2.5%	2.5%

aTIV, adjuvanted trivalent influenza vaccine; DSA, deterministic sensitivity analysis; QIV, quadrivalent influenza vaccine.

Supplementary Figure S7. 2017–2018 DSA Outpatient Visits

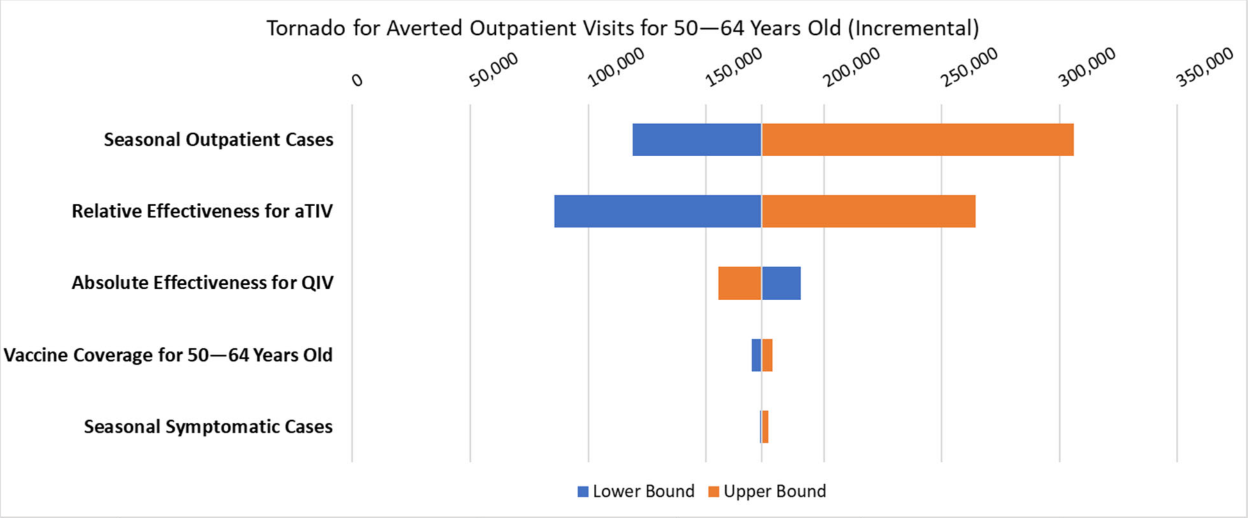

Rank	Parameter Name	Lower Bound: Outpatient Visits Averted	Upper Bound: Outpatient Visits Averted	Lower Bound: Percentage Change From Base Case	Upper Bound: Percentage Change From Base Case
	Base Case	173,759			
1	Seasonal Outpatient Cases	118,924	306,110	-31.6%	76.2%
2	Relative Effectiveness for aTIV	85,649	264,442	-50.7%	52.2%
3	Absolute Effectiveness for QIV	190,347	155,305	9.5%	-10.6%
4	Vaccine Coverage for 50—64 Years Old	169,391	178,174	-2.5%	2.5%
5	Seasonal Symptomatic Cases	172,802	176,542	-0.6%	1.6%

aTIV, adjuvanted trivalent influenza vaccine; DSA, deterministic sensitivity analysis; QIV, quadrivalent influenza vaccine.

Supplementary Figure S8. 2017–2018 DSA Hospitalizations

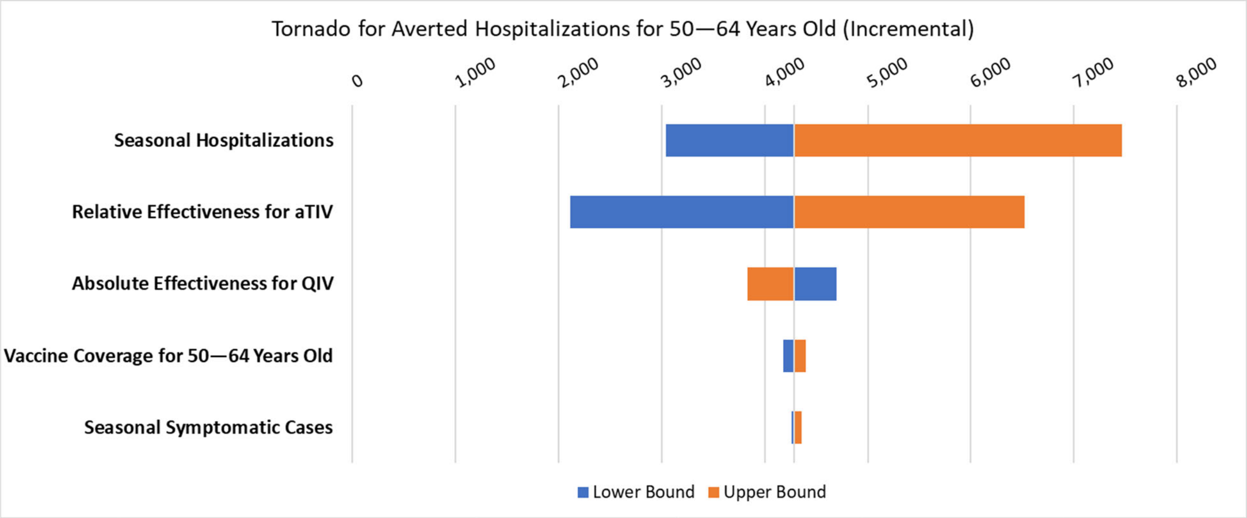

Rank	Parameter Name	Lower Bound: Hospitalizations Averted	Upper Bound: Hospitalizations Averted	Lower Bound: Percentage Change From Base Case	Upper Bound: Percentage Change From Base Case
	Base Case	4,285			
1	Seasonal Hospitalizations	3,043	7,466	-29.0%	74.2%
2	Relative Effectiveness for aTIV	2,112	6,522	-50.7%	52.2%
3	Absolute Effectiveness for QIV	4,694	3,830	9.5%	-10.6%
4	Vaccine Coverage for 50—64 Years Old	4,178	4,394	-2.5%	2.5%
5	Seasonal Symptomatic Cases	4,262	4,354	-0.6%	1.6%

aTIV, adjuvanted trivalent influenza vaccine; DSA, deterministic sensitivity analysis; QIV, quadrivalent influenza vaccine.

Supplementary Figure S9. 2017–2018 DSA ICU Admissions

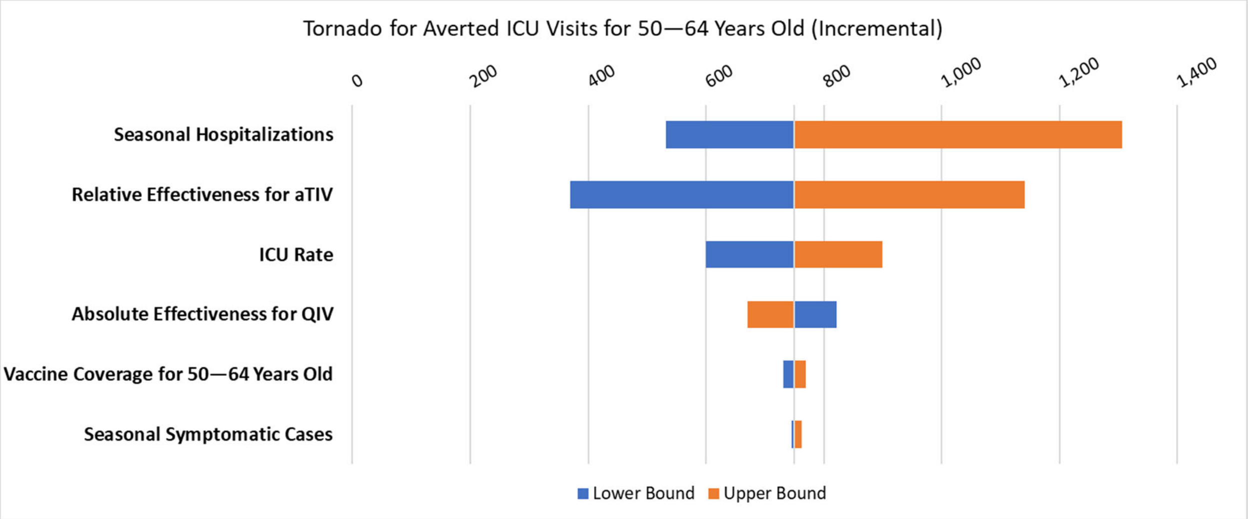

Rank	Parameter Name	Lower Bound: ICU Visits Averted	Upper Bound: ICU Visits Averted	Lower Bound: Percentage Change From Base Case	Upper Bound: Percentage Change From Base Case
	Base Case	750			
1	Seasonal Hospitalizations	533	1,307	-29.0%	74.2%
2	Relative Effectiveness for aTIV	370	1,141	-50.7%	52.2%
3	ICU Rate	600	900	-20.0%	20.0%
4	Absolute Effectiveness for QIV	822	670	9.5%	-10.6%
5	Vaccine Coverage for 50—64 Years Old	731	769	-2.5%	2.5%
6	Seasonal Symptomatic Cases	746	762	-0.6%	1.6%

aTIV, adjuvanted trivalent influenza vaccine; DSA, deterministic sensitivity analysis; ICU, intensive care unit; QIV, quadrivalent influenza vaccine.

Supplementary Figure S10. 2017–2018 DSA Deaths

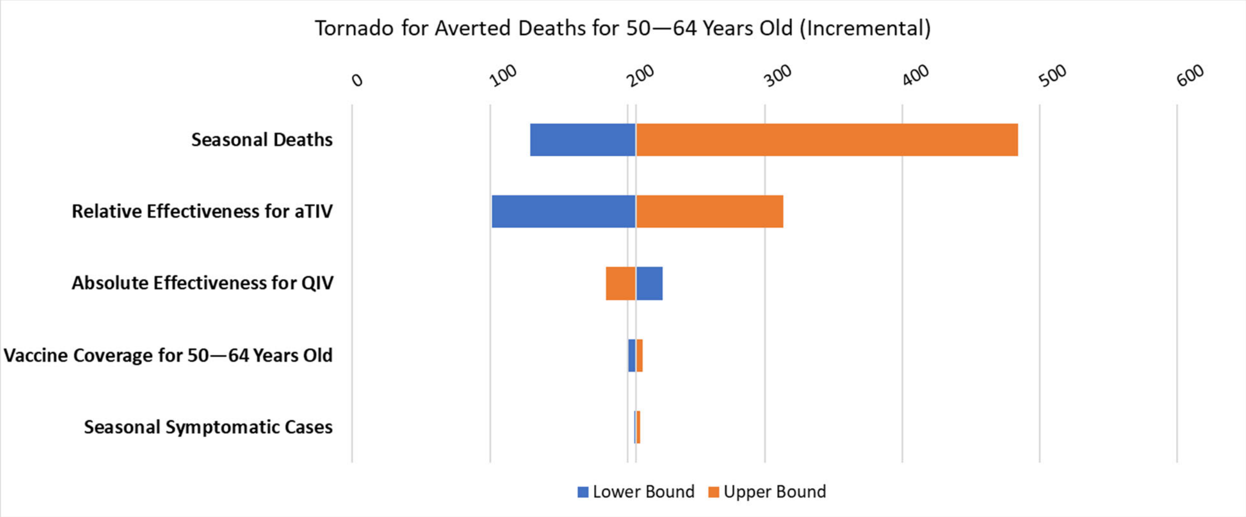

Rank	Parameter Name	Lower Bound: Deaths Averted	Upper Bound: Deaths Averted	Lower Bound: Percentage Change From Base Case	Upper Bound: Percentage Change From Base Case
	Base Case	206			
1	Seasonal Deaths	130	484	-37.1%	135.0%
2	Relative Effectiveness for aTIV	102	314	-50.7%	52.2%
3	Absolute Effectiveness for QIV	226	184	9.5%	-10.6%
4	Vaccine Coverage for 50—64 Years Old	201	211	-2.5%	2.5%
5	Seasonal Symptomatic Cases	205	209	-0.6%	1.6%

aTIV, adjuvanted trivalent influenza vaccine; DSA, deterministic sensitivity analysis; QIV, quadrivalent influenza vaccine.

Supplementary Figure S11. 2018–2019 DSA Symptomatic Illnesses

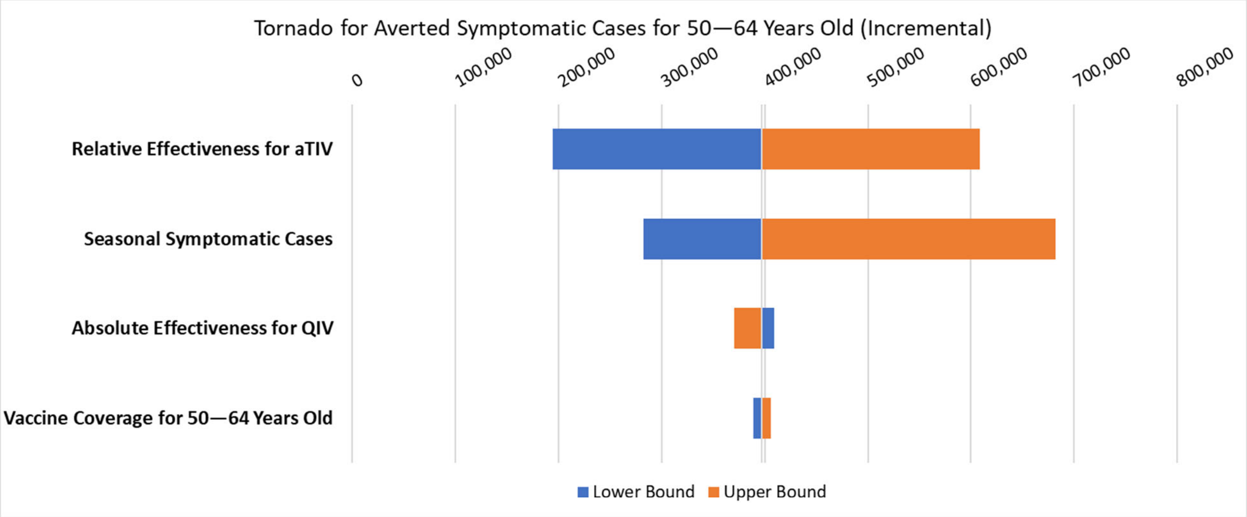

Rank	Parameter Name	Lower Bound: Symptomatic Cases Averted	Upper Bound: Symptomatic Cases Averted	Lower Bound: Percentage Change From Base Case	Upper Bound: Percentage Change From Base Case
	Base Case	397,202			
1	Relative Effectiveness for aTIV	194,560	608,450	-51.0%	53.2%
2	Seasonal Symptomatic Cases	282,383	681,642	-28.9%	71.6%
3	Absolute Effectiveness for QIV	408,887	370,053	2.9%	-6.8%
4	Vaccine Coverage for 50—64 Years Old	388,750	405,706	-2.1%	2.1%

aTIV, adjuvanted trivalent influenza vaccine; DSA, deterministic sensitivity analysis; QIV, quadrivalent influenza vaccine.

Supplementary Figure S12. 2018–2019 DSA Deaths

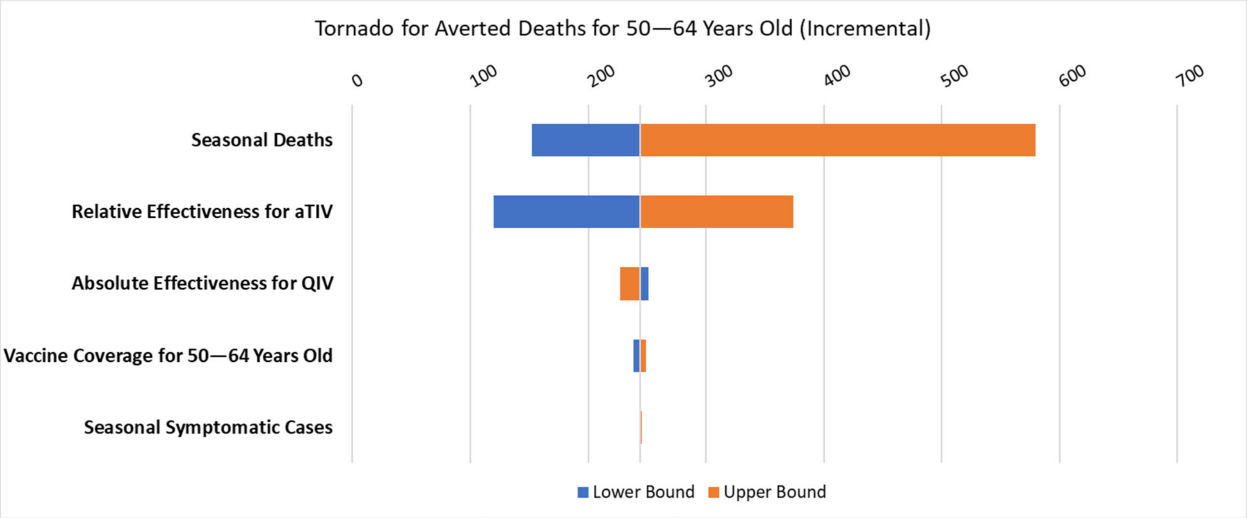

Rank	Parameter Name	Lower Bound: Deaths Averted	Upper Bound: Deaths Averted	Lower Bound: Percentage Change From Base Case	Upper Bound: Percentage Change From Base Case
	Base Case	244			
1	Seasonal Deaths	153	580	-37.5%	137.6%
2	Relative Effectiveness for aTIV	120	374	-51.0%	53.2%
3	Absolute Effectiveness for QIV	251	227	2.9%	-6.8%
4	Vaccine Coverage for 50—64 Years Old	239	249	-2.1%	2.1%
5	Seasonal Symptomatic Cases	243	246	-0.2%	0.6%

aTIV, adjuvanted trivalent influenza vaccine; DSA, deterministic sensitivity analysis; QIV, quadrivalent influenza vaccine.

Supplementary Figure S13. 2019–2020 DSA Symptomatic Illnesses

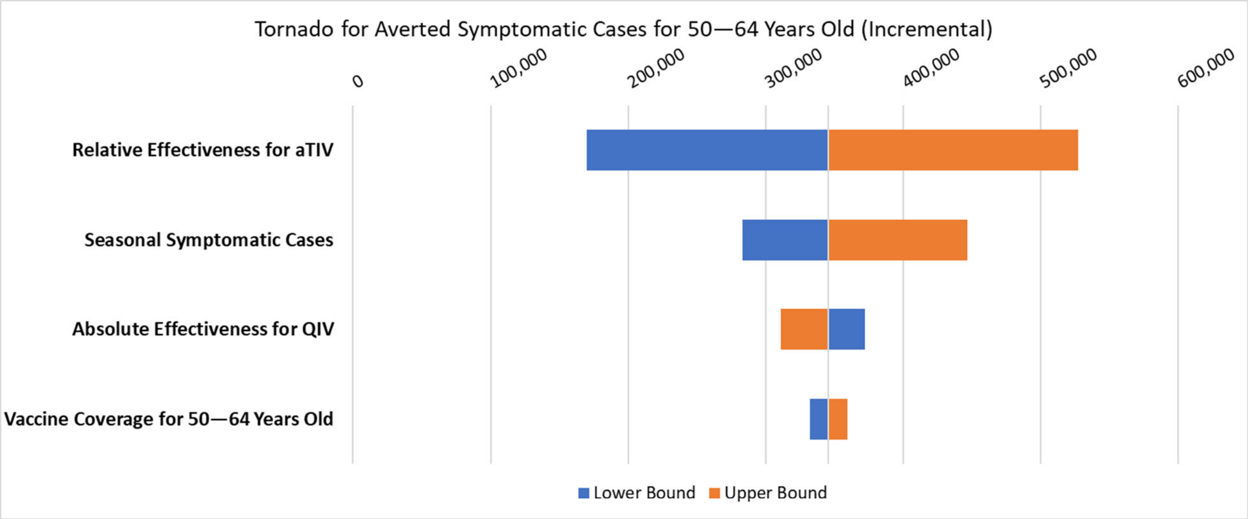

Rank	Parameter Name	Lower Bound: Symptomatic Cases Averted	Upper Bound: Symptomatic Cases Averted	Lower Bound: Percentage Change From Base Case	Upper Bound: Percentage Change From Base Case
	Base Case	345,506			
1	Relative Effectiveness for aTIV	169,766	527,548	-50.9%	52.7%
2	Seasonal Symptomatic Cases	283,229	446,810	-18.0%	29.3%
3	Absolute Effectiveness for QIV	372,420	310,801	7.8%	-10.0%
4	Vaccine Coverage for 50—64 Years Old	332,020	359,324	-3.9%	4.0%

aTIV, adjuvanted trivalent influenza vaccine; DSA, deterministic sensitivity analysis; QIV, quadrivalent influenza vaccine.

Supplementary Figure S14. 2019–2020 DSA Deaths

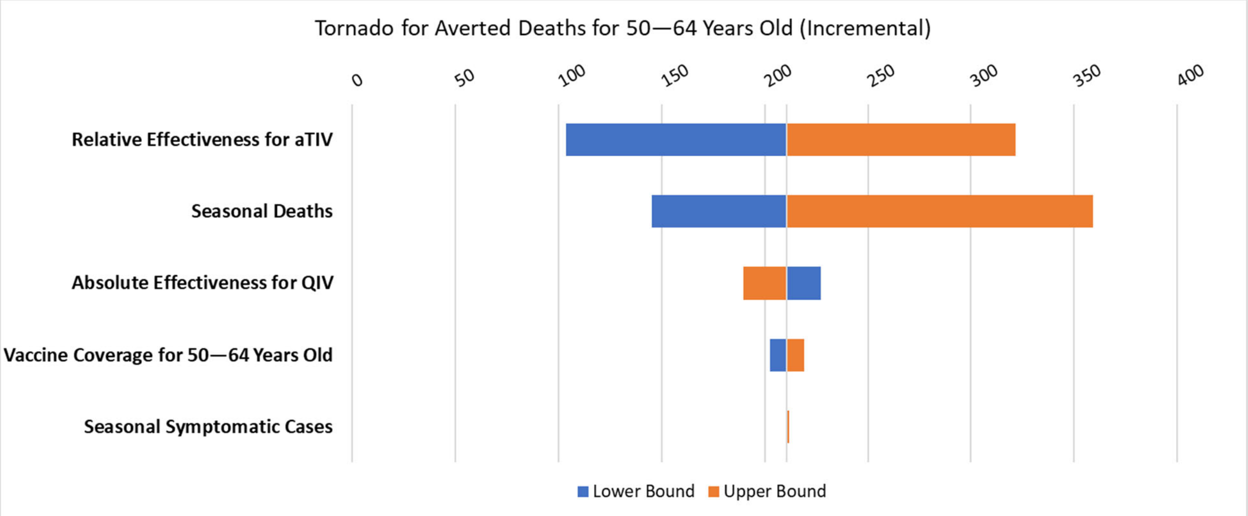

Rank	Parameter Name	Lower Bound: Deaths Averted	Upper Bound: Deaths Averted	Lower Bound: Percentage Change From Base Case	Upper Bound: Percentage Change From Base Case
	Base Case	211			
1	Relative Effectiveness for aTIV	104	322	-50.9%	52.7%
2	Seasonal Deaths	145	359	-31.2%	70.5%
3	Absolute Effectiveness for QIV	227	190	7.8%	-10.0%
4	Vaccine Coverage for 50—64 Years Old	202	219	-3.9%	4.0%
5	Seasonal Symptomatic Cases	210	212	-0.3%	0.5%

aTIV, adjuvanted trivalent influenza vaccine; DSA, deterministic sensitivity analysis; QIV, quadrivalent influenza vaccine.

Supplementary Figure S15. 2022–2023 DSA Symptomatic Illnesses

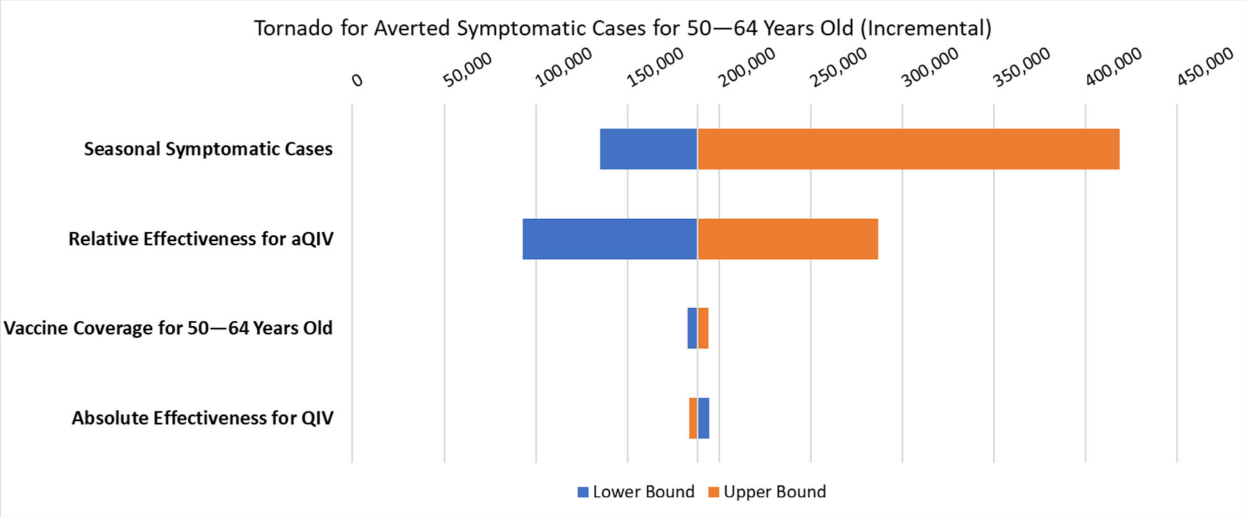

Rank	Parameter Name	Lower Bound: Symptomatic Cases Averted	Upper Bound: Symptomatic Cases Averted	Lower Bound: Percentage Change From Base Case	Upper Bound: Percentage Change From Base Case
	Base Case	188,467			
1	Seasonal Symptomatic Cases	135,119	418,648	-28.3%	122.1%
2	Relative Effectiveness for aQIV	92,894	286,844	-50.7%	52.2%
3	Vaccine Coverage for 50—64 Years Old	182,987	194,046	-2.9%	3.0%
4	Absolute Effectiveness for QIV	194,479	183,559	3.2%	-2.6%

aQIV, adjuvanted quadrivalent influenza vaccine; DSA, deterministic sensitivity analysis; QIV, quadrivalent influenza vaccine.

Supplementary Figure S16. 2022–2023 DSA Deaths

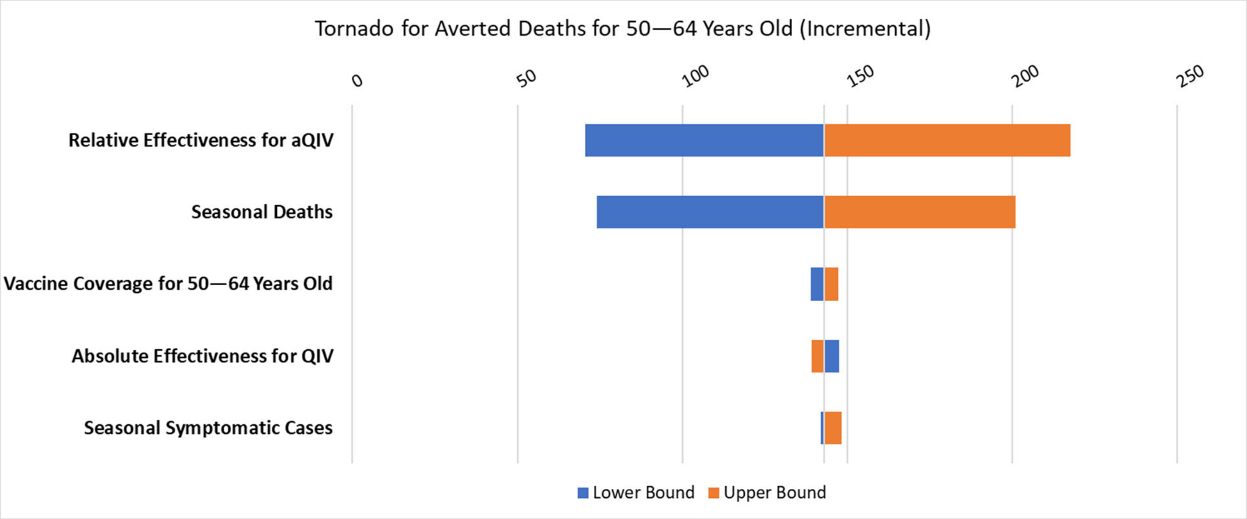

Rank	Parameter Name	Lower Bound: Deaths Averted	Upper Bound: Deaths Averted	Lower Bound: Percentage Change From Base Case	Upper Bound: Percentage Change From Base Case
	Base Case	143			
1	Relative Effectiveness for aQIV	70	218	-50.7%	52.2%
2	Seasonal Deaths	74	201	-48.2%	40.5%
3	Vaccine Coverage for 50—64 Years Old	139	147	-2.9%	3.0%
4	Absolute Effectiveness for QIV	148	139	3.2%	-2.6%
5	Seasonal Symptomatic Cases	142	148	-0.8%	3.6%

aQIV, adjuvanted quadrivalent influenza vaccine; DSA, deterministic sensitivity analysis; QIV, quadrivalent influenza vaccine.

Supplementary Figure S17. 2023–2024 DSA Symptomatic Illnesses

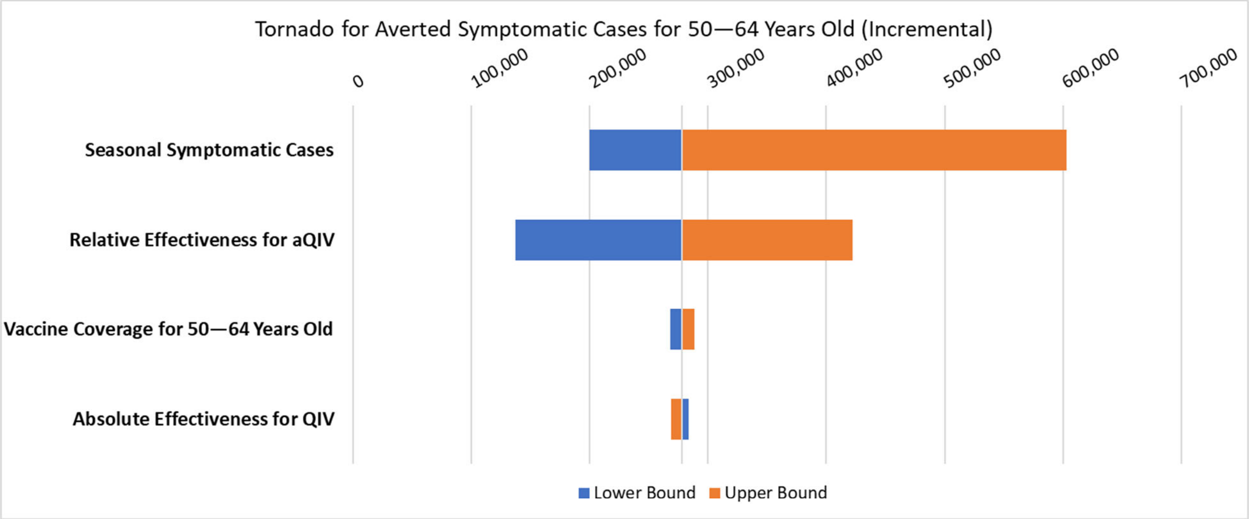

Rank	Parameter Name	Lower Bound: Symptomatic Cases Averted	Upper Bound: Symptomatic Cases Averted	Lower Bound: Percentage Change From Base Case	Upper Bound: Percentage Change From Base Case
	Base Case	277,943			
1	Seasonal Symptomatic Cases	199,456	603,336	-28.2%	117.1%
2	Relative Effectiveness for aQIV	137,154	422,521	-50.7%	52.0%
3	Vaccine Coverage for 50—64 Years Old	267,689	288,459	-3.7%	3.8%
4	Absolute Effectiveness for QIV	283,626	268,863	2.0%	-3.3%

aQIV, adjuvanted quadrivalent influenza vaccine; DSA, deterministic sensitivity analysis; QIV, quadrivalent influenza vaccine.

Supplementary Figure S18. 2023–2024 DSA Deaths

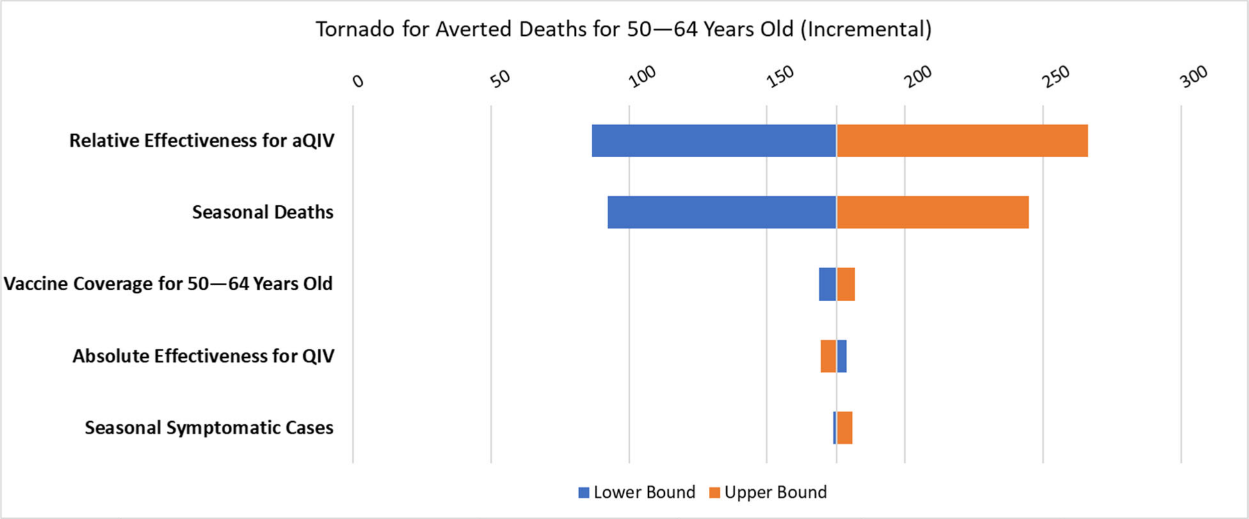

Rank	Parameter Name	Lower Bound: Deaths Averted	Upper Bound: Deaths Averted	Lower Bound: Percentage Change From Base Case	Upper Bound: Percentage Change From Base Case
	Base Case	175			
1	Relative Effectiveness for aQIV	86	266	-50.7%	52.0%
2	Seasonal Deaths	92	245	-47.4%	39.9%
3	Vaccine Coverage for 50—64 Years Old	169	182	-3.7%	3.8%
4	Absolute Effectiveness for QIV	179	170	2.0%	-3.3%
5	Seasonal Symptomatic Cases	174	181	-0.7%	3.3%

aQIV, adjuvanted quadrivalent influenza vaccine; DSA, deterministic sensitivity analysis; QIV, quadrivalent influenza vaccine.

Supplementary Figure S19. 2017–2018 PSA Symptomatic Cases

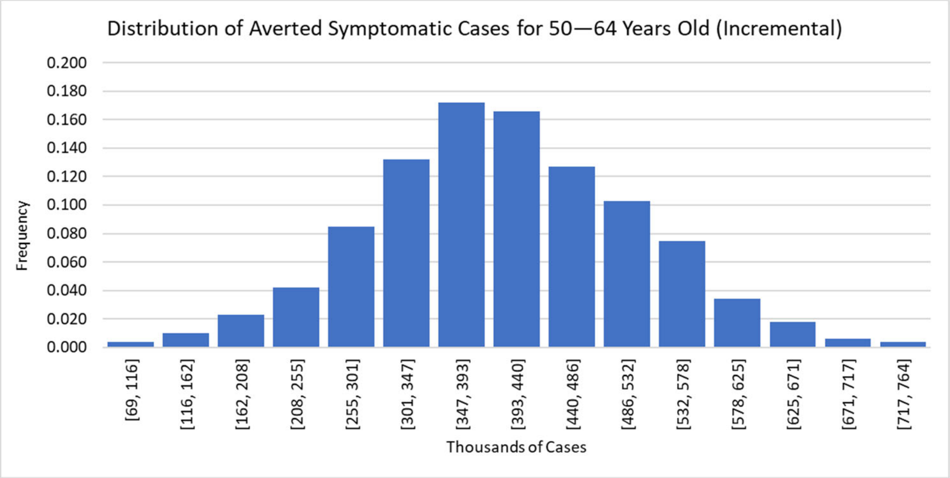

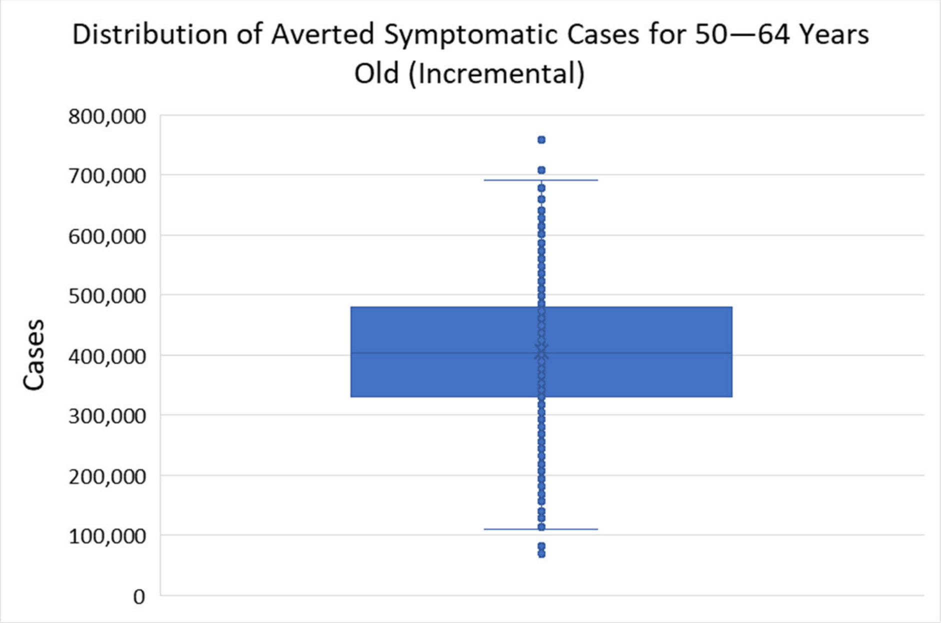

Scenario	Mean	First Quartile	Third Quartile
aTIV	1,953,236	1,446,177	2,364,882
QIV	1,547,154	1,068,666	1,918,463
Incremental	406,082	331,339	480,552

aTIV, adjuvanted trivalent influenza vaccine; PSA, probabilistic sensitivity analysis; QIV, quadrivalent influenza vaccine.

Supplementary Figure S20. 2017–2018 PSA Outpatient Visits

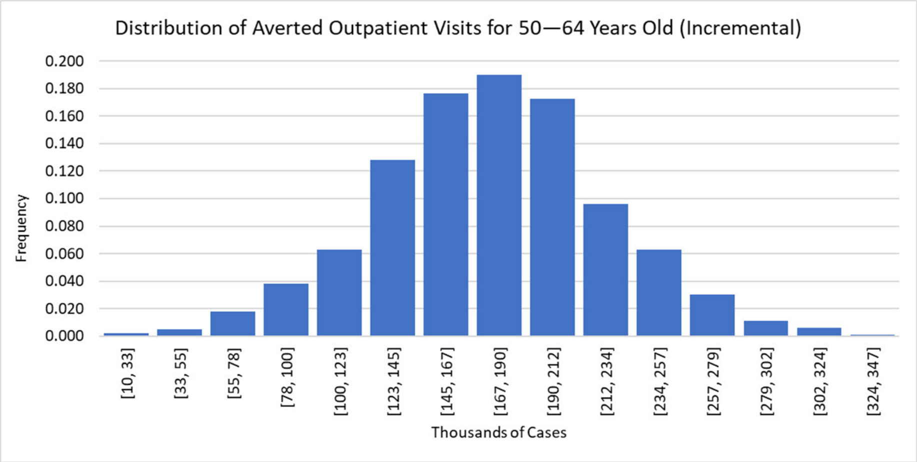

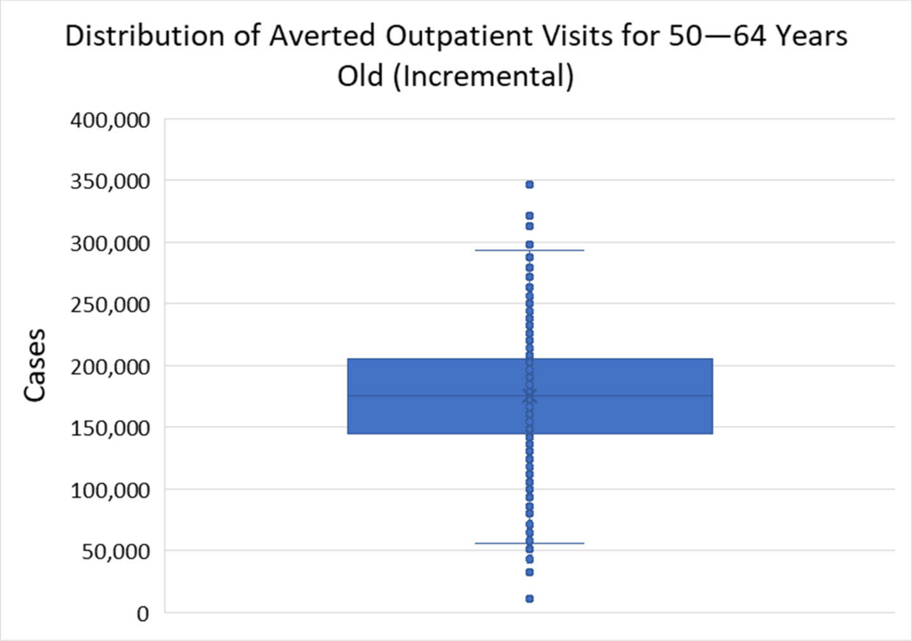

Scenario	Mean	First Quartile	Third Quartile
aTIV	840,672	641,809	1,015,044
QIV	665,260	476,722	822,681
Incremental	175,411	144,642	205,674

aTIV, adjuvanted trivalent influenza vaccine; PSA, probabilistic sensitivity analysis; QIV, quadrivalent influenza vaccine.

Supplementary Figure S21. 2017–2018 PSA Hospitalizations

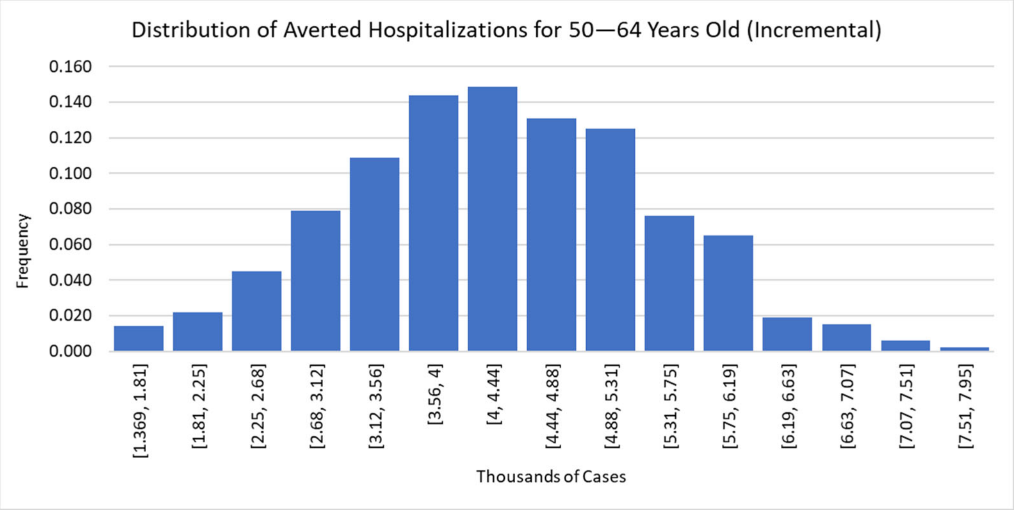

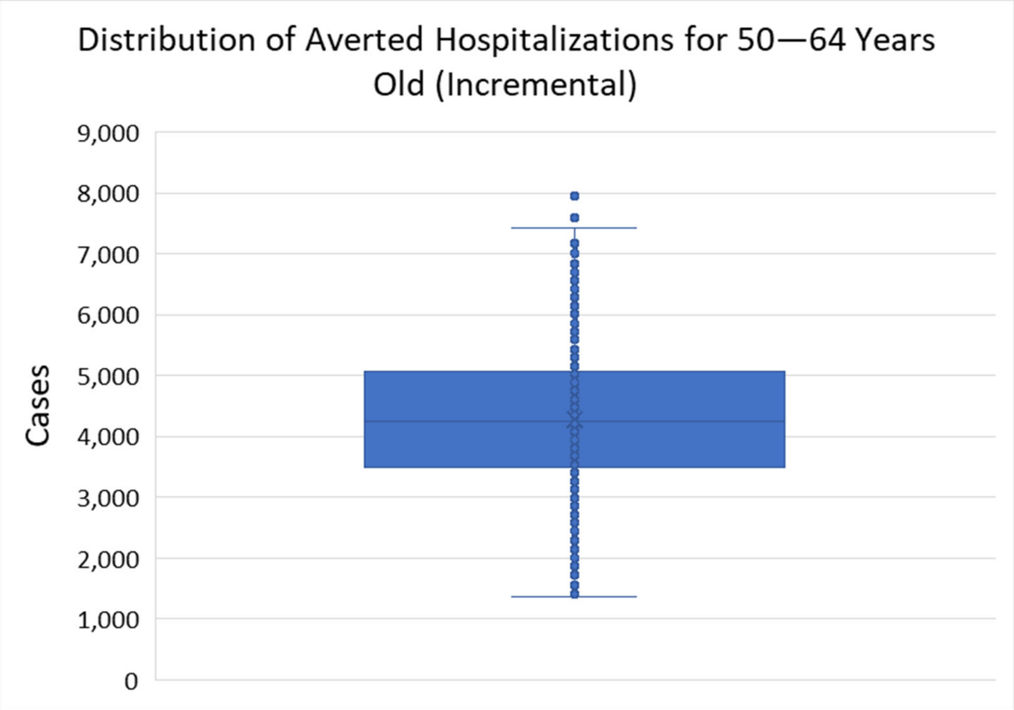

Scenario	Mean	First Quartile	Third Quartile
aTIV	20,550	15,461	24,600
QIV	16,272	11,570	19,899
Incremental	4277	3491	5064

aTIV, adjuvanted trivalent influenza vaccine; PSA, probabilistic sensitivity analysis; QIV, quadrivalent influenza vaccine.

Supplementary Figure S22. 2017–2018 PSA ICU Admissions

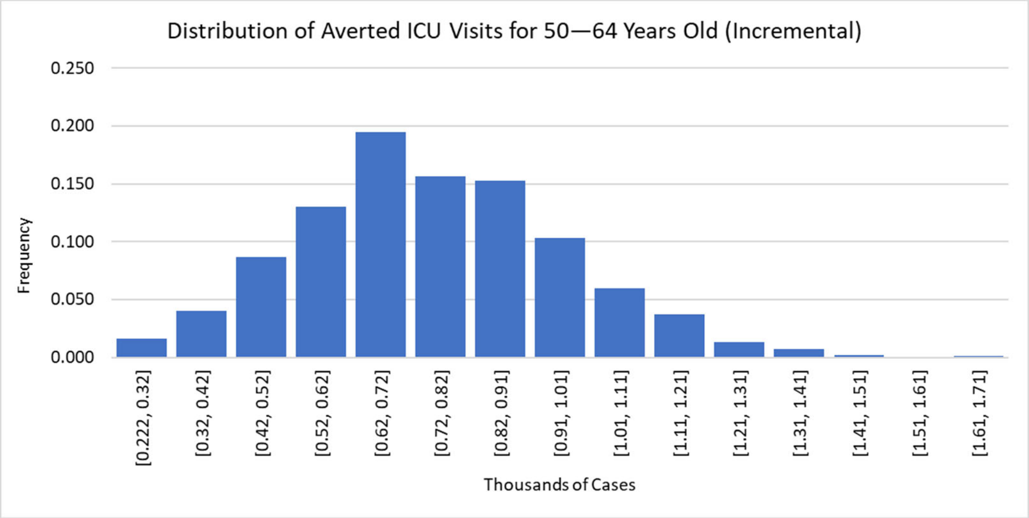

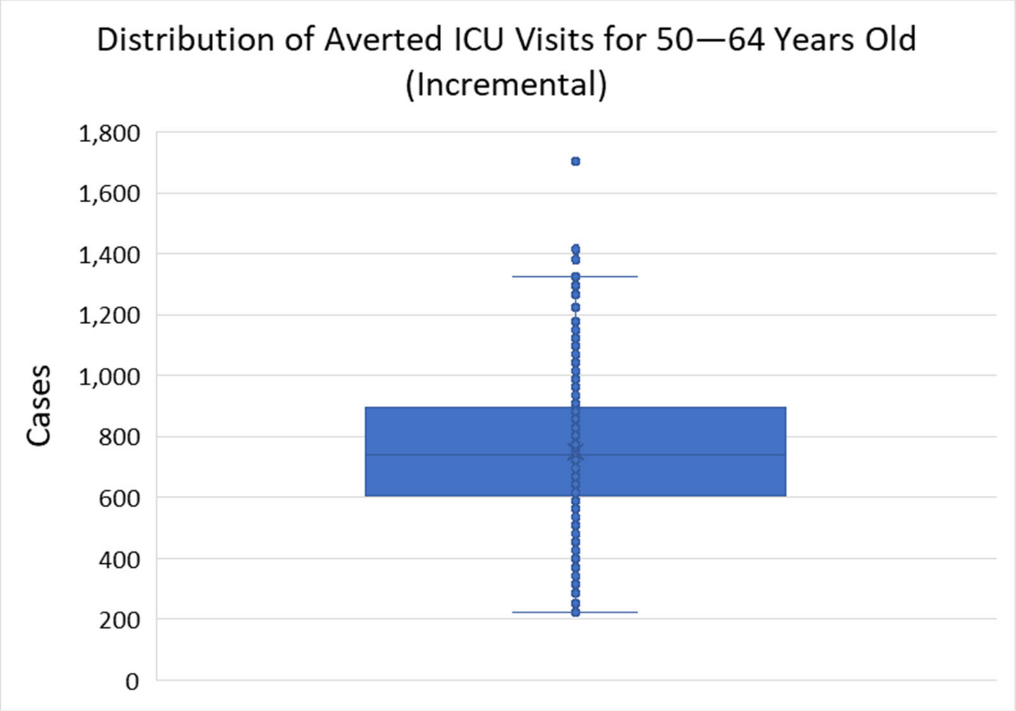

Scenario	Mean	First Quartile	Third Quartile
aTIV	3603	2628	4384
QIV	2852	1986	3529
Incremental	751	606	893

aTIV, adjuvanted trivalent influenza vaccine; PSA, probabilistic sensitivity analysis; QIV, quadrivalent influenza vaccine.

Supplementary Figure S23. 2017–2018 PSA Deaths

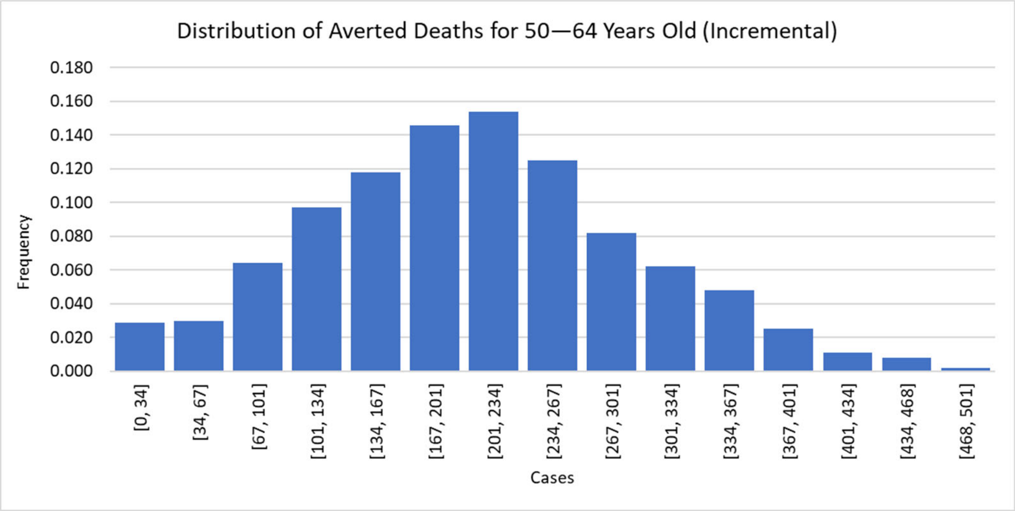

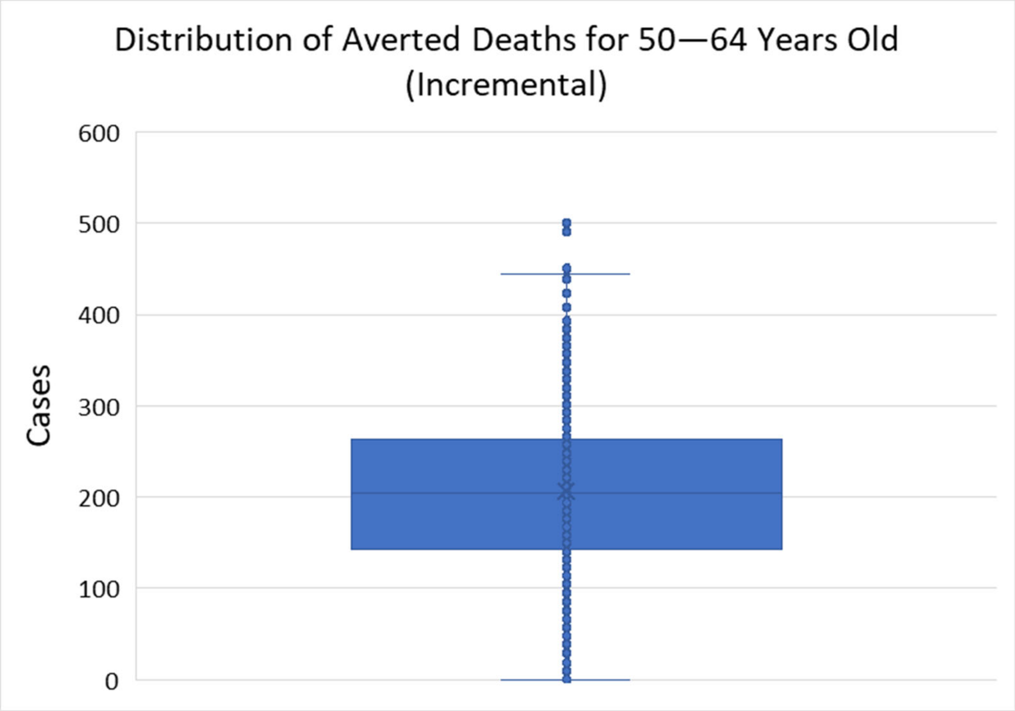

**Scenario**	**Mean**	**First Quartile**	**Third Quartile**
aTIV	993	660	1289
QIV	786	493	1029
Incremental	206	143	264

aTIV, adjuvanted trivalent influenza vaccine; PSA, probabilistic sensitivity analysis; QIV, quadrivalent influenza vaccine.

Supplementary Figure S24. 2018–2019 PSA Symptomatic Illnesses

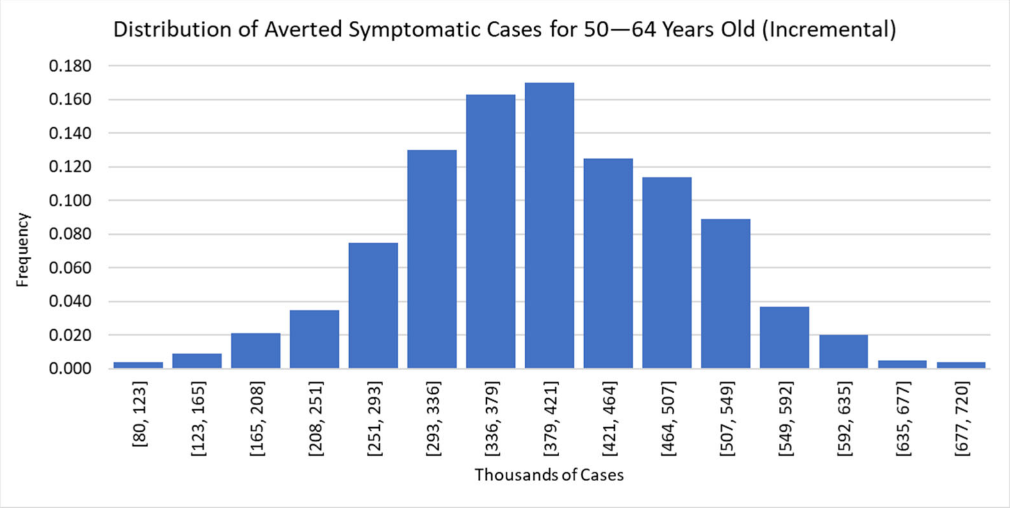

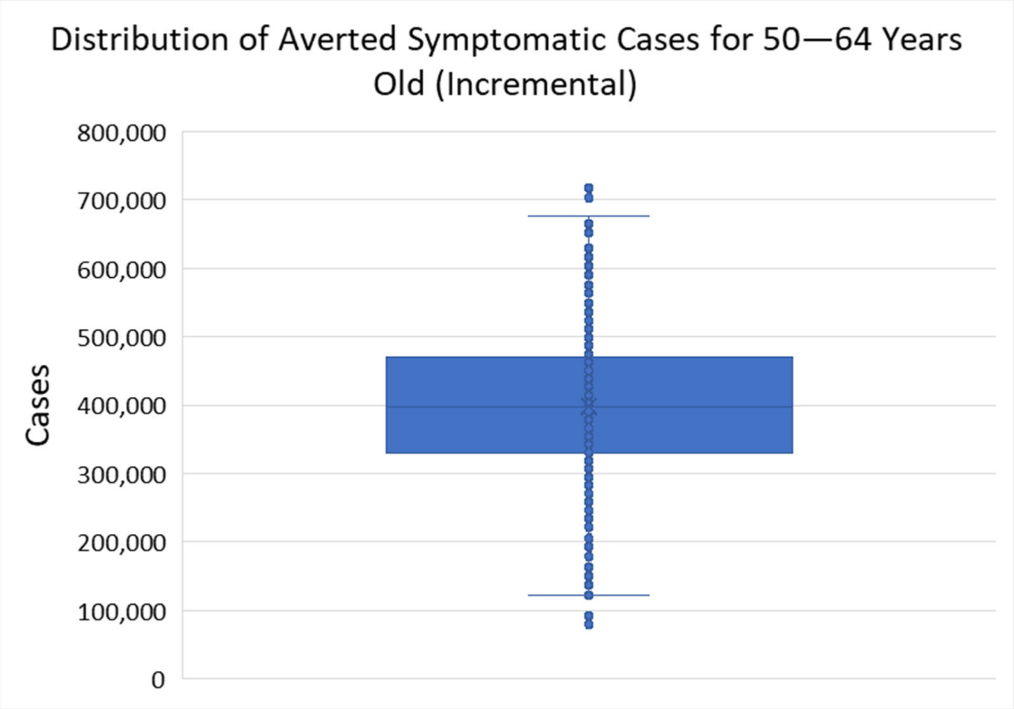

Scenario	Mean	First Quartile	Third Quartile
aTIV	1,024,987	594,082	1,275,955
QIV	626,949	210,921	853,041
Incremental	398,037	329,762	470,168

aTIV, adjuvanted trivalent influenza vaccine; PSA, probabilistic sensitivity analysis; QIV, quadrivalent influenza vaccine.

Supplementary Figure S25. 2018–2019 PSA Deaths

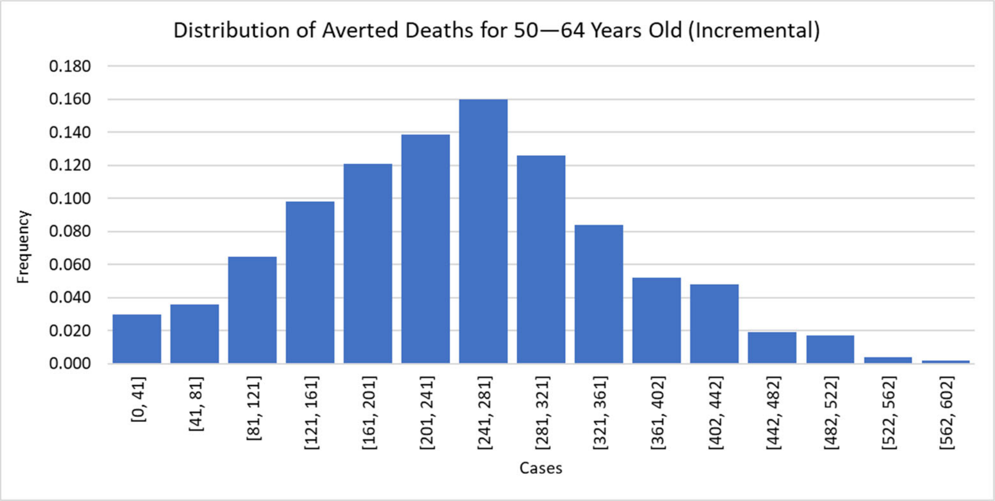

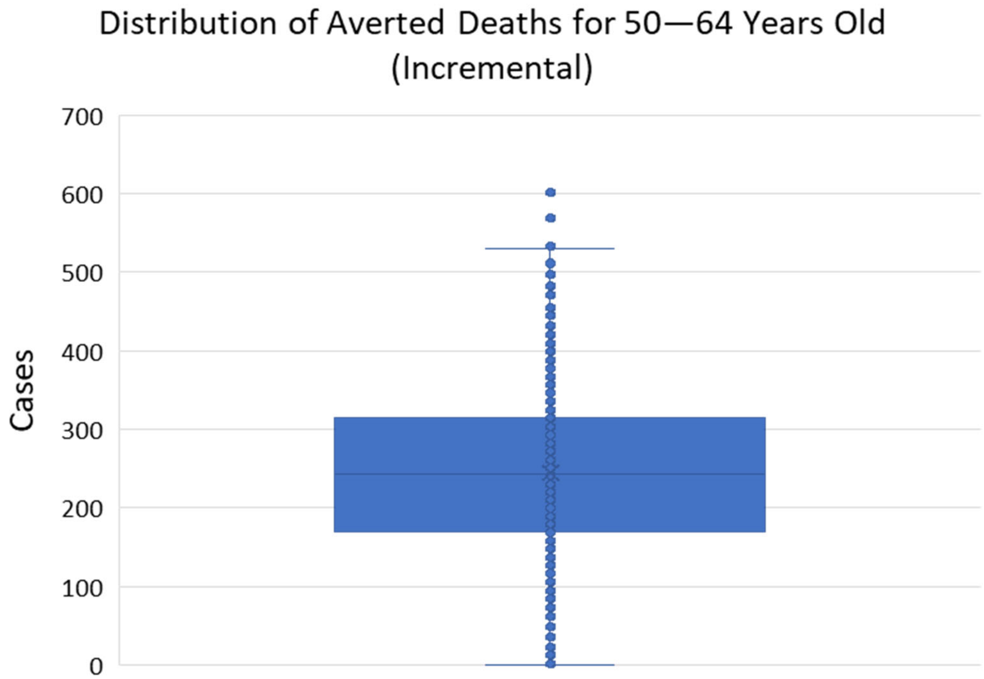

Scenario	Mean	First Quartile	Third Quartile
aTIV	629	333	811
QIV	385	114	516
Incremental	244	169	314

aTIV, adjuvanted trivalent influenza vaccine; PSA, probabilistic sensitivity analysis; QIV, quadrivalent influenza vaccine.

Supplementary Figure S26. 2019–2020 PSA Symptomatic Illnesses

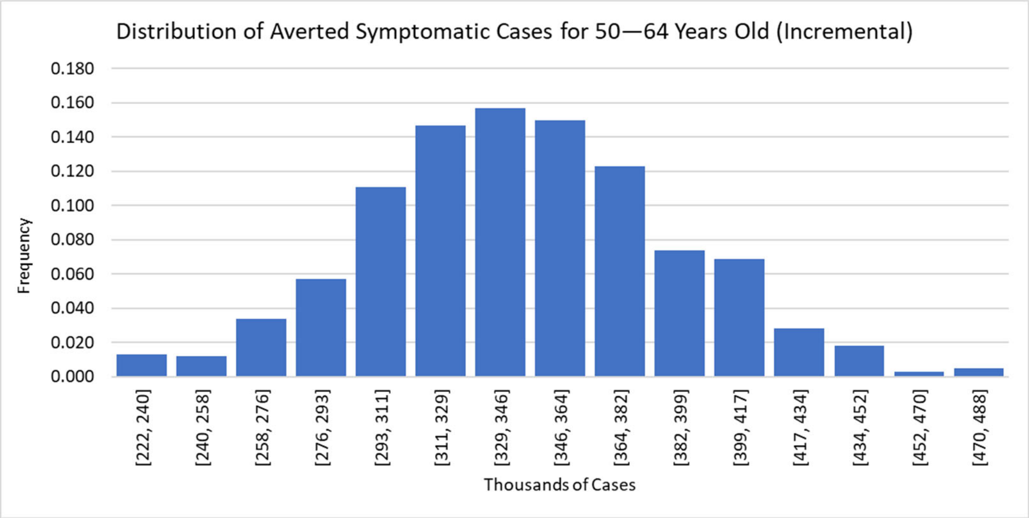

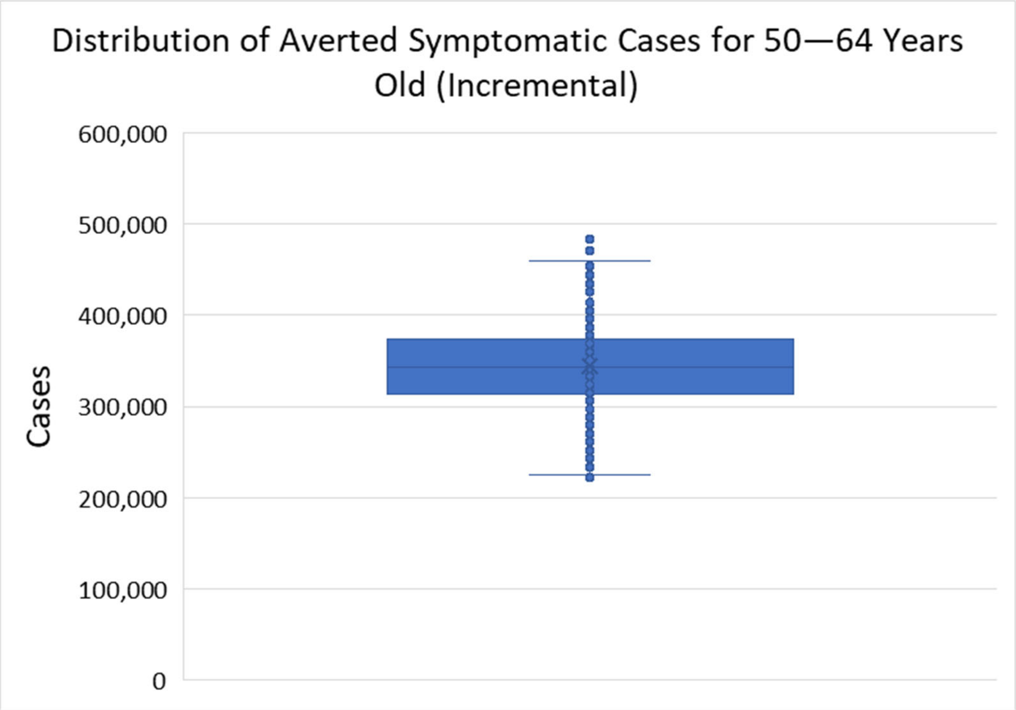

Scenario	Mean	First Quartile	Third Quartile
**aTIV**	2,201,317	1,816,336	2,537,487
**QIV**	1,856,910	1,474,137	2,177,918
**Incremental**	344,407	313,617	373,143

aTIV, adjuvanted trivalent influenza vaccine; PSA, probabilistic sensitivity analysis; QIV, quadrivalent influenza vaccine.

Supplementary Figure S27. 2019–2020 PSA Deaths

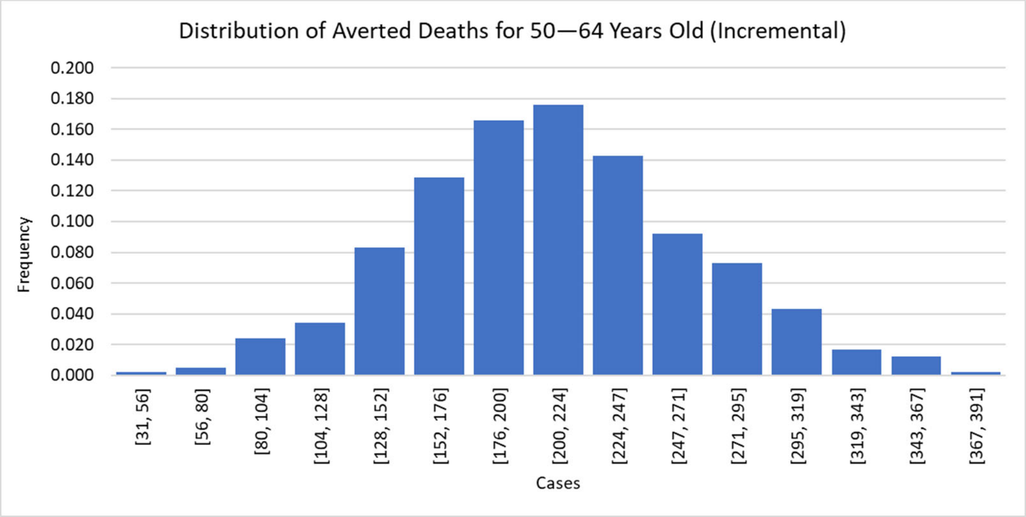

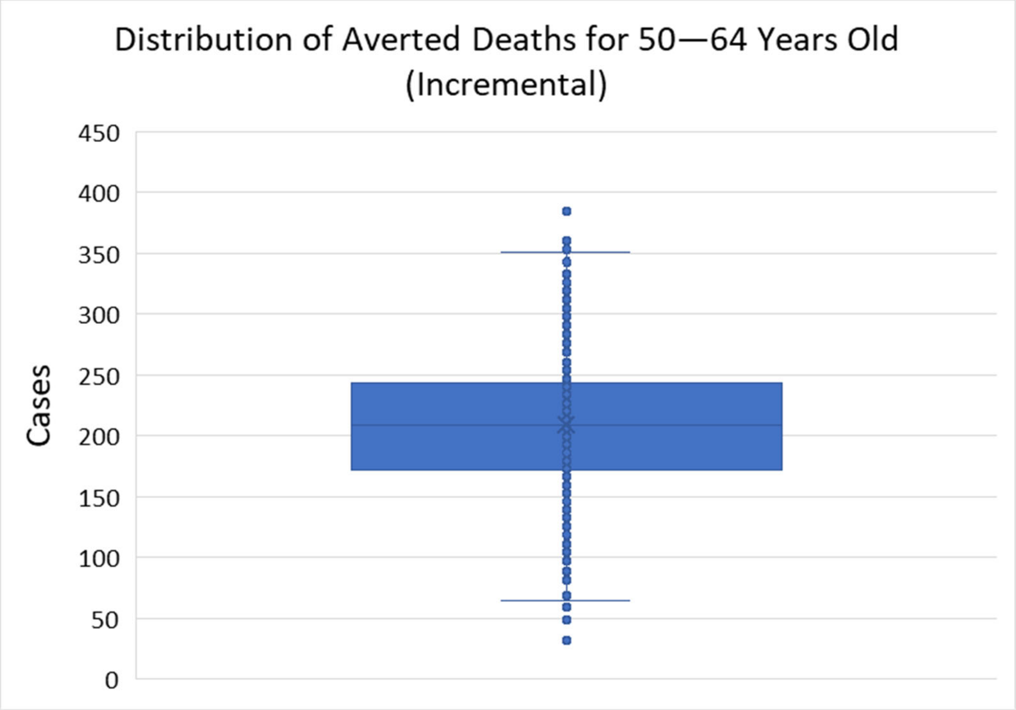

**Scenario**	**Mean**	**First Quartile**	**Third Quartile**
**aTIV**	1339	1029	1617
**QIV**	1130	845	1373
**Incremental**	210	172	244

aTIV, adjuvanted trivalent influenza vaccine; PSA, probabilistic sensitivity analysis; QIV, quadrivalent influenza vaccine.

Supplementary Figure S28. 2022–2023 PSA Symptomatic Illnesses

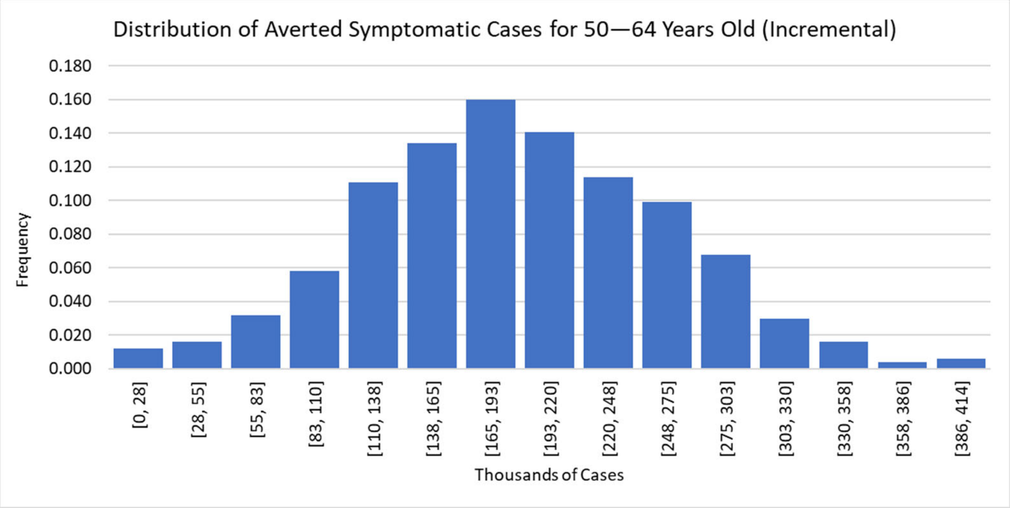

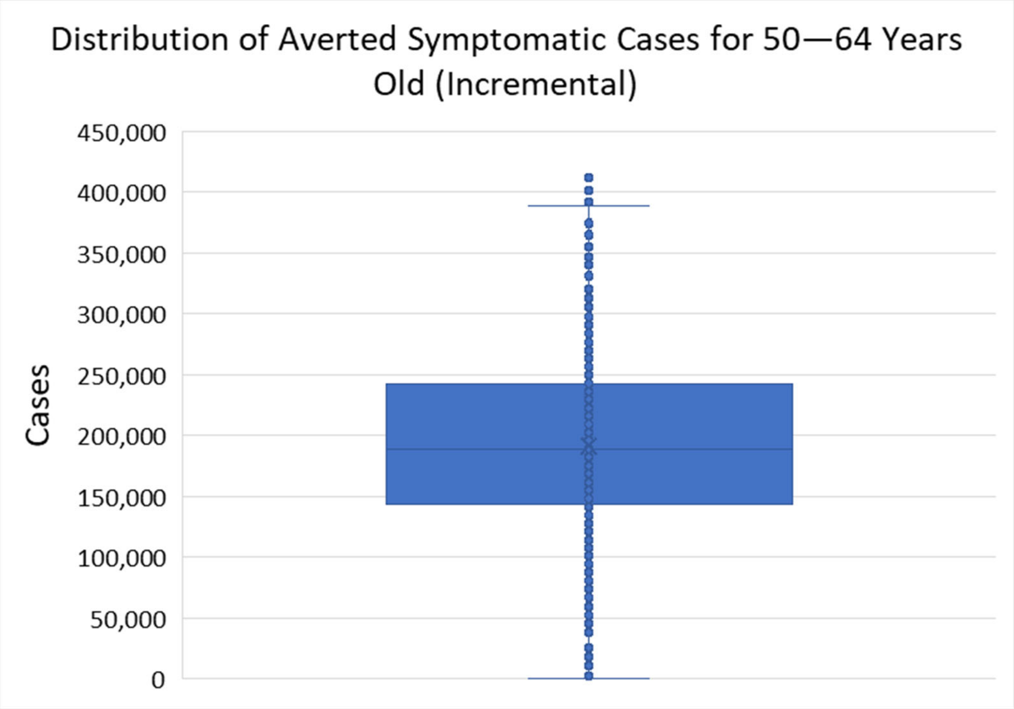

Scenario	Mean	First Quartile	Third Quartile
**aTIV**	1,435,604	1,060,709	1,809,967
**QIV**	1,244,132	920,270	1,569,304
**Incremental**	191,472	143,196	241,950

aTIV, adjuvanted trivalent influenza vaccine; PSA, probabilistic sensitivity analysis; QIV, quadrivalent influenza vaccine.

Supplementary Figure S29. 2022–2023 PSA Deaths

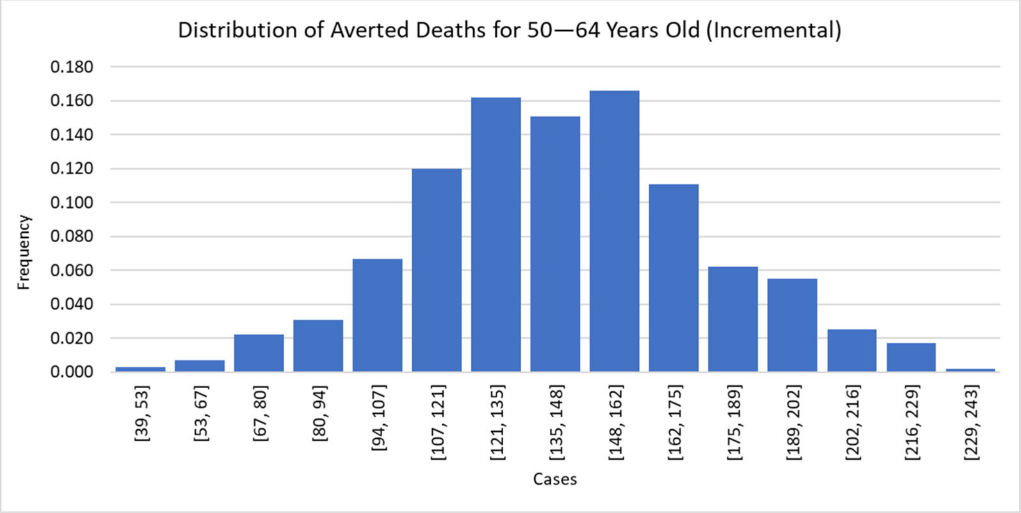

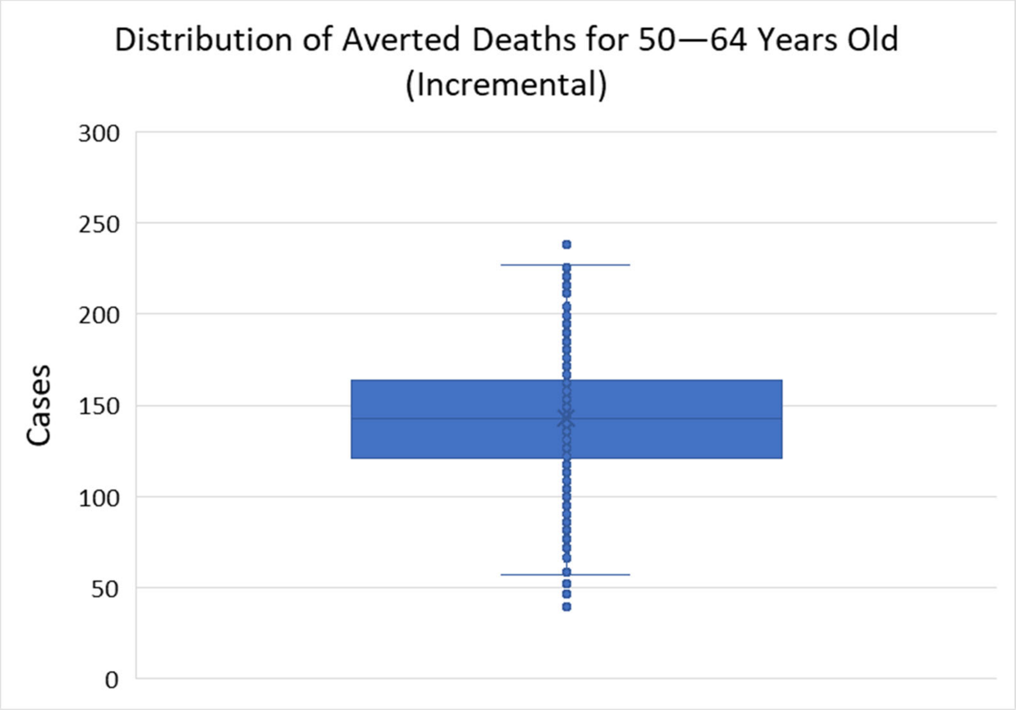

**Scenario**	**Mean**	**First Quartile**	**Third Quartile**
**aTIV**	1074	910	1239
**QIV**	931	790	1076
**Incremental**	143	121	164

aTIV, adjuvanted trivalent influenza vaccine; PSA, probabilistic sensitivity analysis; QIV, quadrivalent influenza vaccine.

Supplementary Figure S30. 2023–2024 PSA Symptomatic Illnesses

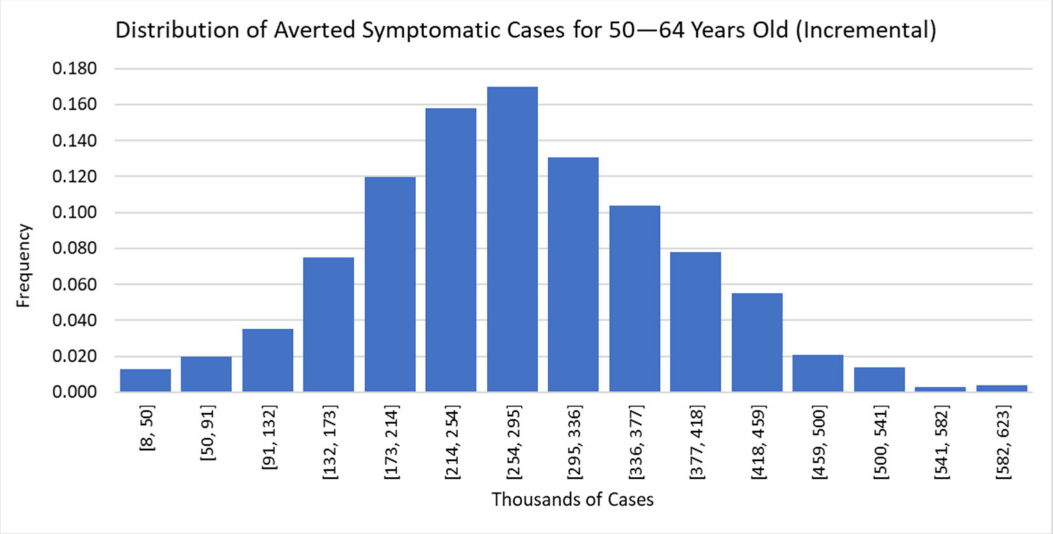

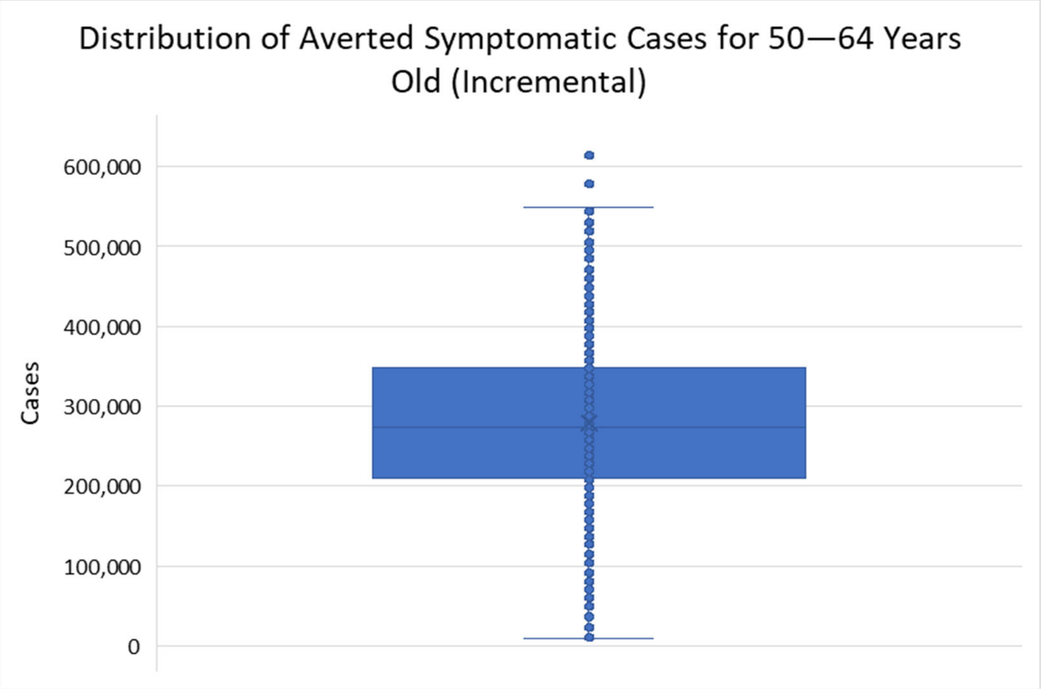

**Scenario**	**Mean**	**First Quartile**	**Third Quartile**
**aQIV**	2,644,388	1,900,953	3,339,034
**QIV**	2,365,183	1,679,615	2,986,464
**Incremental**	279,206	210,271	347,999

aQIV, adjuvanted quadrivalent influenza vaccine; PSA, probabilistic sensitivity analysis; QIV, quadrivalent influenza vaccine.

Supplementary Figure S31. 2023–2024 PSA Deaths

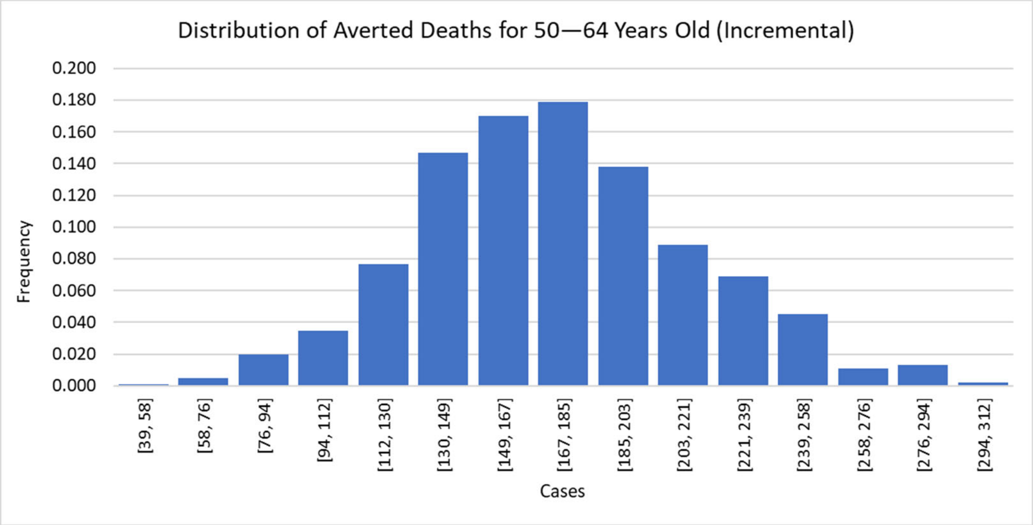

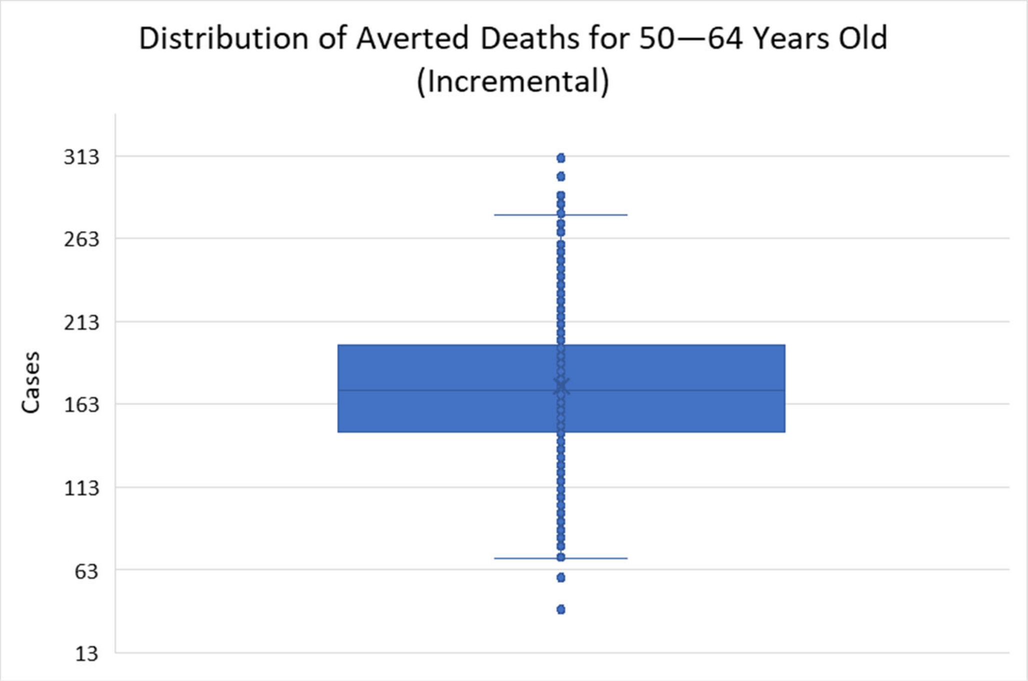

Scenario	Mean	First Quartile	Third Quartile
**aQIV**	1644	1325	1929
**QIV**	1471	1169	1740
**Incremental**	174	146	199

aQIV, adjuvanted quadrivalent influenza vaccine; PSA, probabilistic sensitivity analysis; QIV, quadrivalent influenza vaccine.
